# Predicting lupus membranous nephritis using reduced picolinic acid to tryptophan ratio as a urinary biomarker

**DOI:** 10.1016/j.isci.2021.103355

**Published:** 2021-10-25

**Authors:** Krittima Anekthanakul, Siriphan Manocheewa, Kittiphan Chienwichai, Patcha Poungsombat, Suphitcha Limjiasahapong, Kwanjeera Wanichthanarak, Narumol Jariyasopit, Vivek Bhakta Mathema, Chutima Kuhakarn, Vichai Reutrakul, Jutarop Phetcharaburanin, Atikorn Panya, Natthaporn Phonsatta, Wonnop Visessanguan, Yotsawat Pomyen, Yongyut Sirivatanauksorn, Suchin Worawichawong, Nuankanya Sathirapongsasuti, Chagriya Kitiyakara, Sakda Khoomrung

**Affiliations:** 1 Metabolomics and Systems Biology, Department of Biochemistry, Faculty of Medicine Siriraj Hospital, Mahidol University, Bangkok 10700, Thailand; 2 Siriraj Metabolomics and Phenomics Center, Faculty of Medicine Siriraj Hospital, Mahidol University, Bangkok 10700, Thailand; 3 Department of Medicine, Faculty of Medicine Ramathibodi Hospital, Mahidol University, Bangkok 10400, Thailand; 4 Hatyai hospital, Songkhla 90110, Thailand; 5 Department of Chemistry and Center of Excellence for Innovation in Chemistry (PERCH-CIC), Faculty of Science, Mahidol University, Bangkok 10400, Thailand; 6 Department of Biochemistry, Faculty of Medicine, Khon Kaen University, Khon Kaen 40002, Thailand; 7 Cholangiocarcinoma Research Institute, Khon Kaen University, Khon Kaen 40002, Thailand; 8 Khon Kaen University International Phenome Laboratory, Khon Kaen University, Khon Kaen 40002, Thailand; 9 Functional Ingredients and Food Biotechnology Research Unit, National Center for Genetic Engineering and Biotechnology (BIOTEC), Pathumthani 12120, Thailand; 10 Translational Research Unit, Chulabhorn Research Institute, Bangkok 10210, Thailand; 11 Department of Pathology, Faculty of Medicine Ramathibodi Hospital, Mahidol University, Bangkok 10400, Thailand; 12 Section of Translational Medicine, Faculty of Medicine Ramathibodi Hospital, Mahidol University, Bangkok 10400, Thailand; 13 Research Network of NANOTEC - MU Ramathibodi on Nanomedicine, Bangkok 10400, Thailand

**Keywords:** Biopsy sample, Body substance sample, Metabolomics

## Abstract

The current gold standard for classifying lupus nephritis (LN) progression is a renal biopsy, which is an invasive procedure. Undergoing a series of biopsies for monitoring disease progression and treatments is unlikely suitable for patients with LN. Thus, there is an urgent need for non-invasive alternative biomarkers that can facilitate LN class diagnosis. Such biomarkers will be very useful in guiding intervention strategies to mitigate or treat patients with LN. Urine samples were collected from two independent cohorts. Patients with LN were classified into proliferative (class III/IV) and membranous (class V) by kidney histopathology. Metabolomics was performed to identify potential metabolites, which could be specific for the classification of membranous LN. The ratio of picolinic acid (Pic) to tryptophan (Trp) ([Pic/Trp] ratio) was found to be a promising candidate for LN diagnostic and membranous classification. It has high potential as an alternative biomarker for the non-invasive diagnosis of LN.

## Highlights

• The urinary [Fum], [Ket], [Pyr], and [Mal] are significantly higher in patients with LN
• The urinary [Pic/Trp] ratio is significantly lower in patients with LN
• The [Pic/Trp] is very specific for classifying proliferative LN from membranous LN
• Combining [Pic/Trp] with urine protein–creatinine ratio (UPCR) and estimated glomerular filtration rate (eGFR) further improves membranous LN prediction

## Graphical abstract

**Figure:**
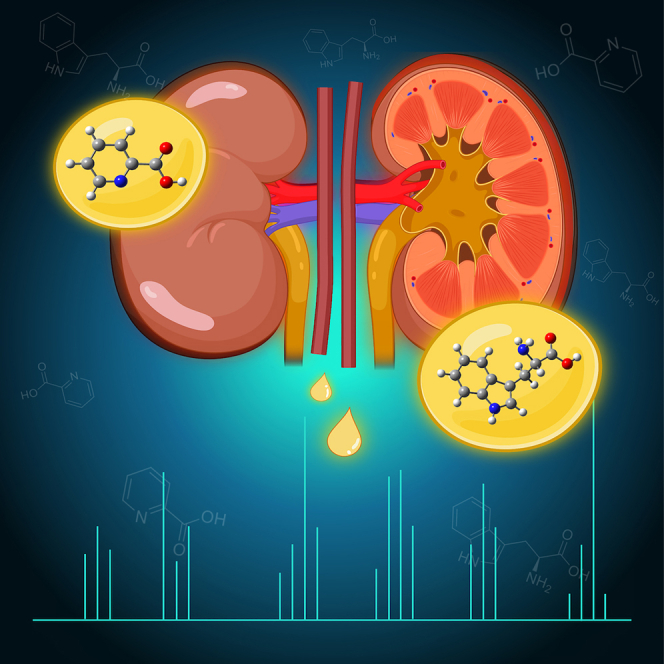

## Introduction

Systemic lupus erythematosus (SLE) is a multi-system autoimmune disease primarily affecting young to middle-aged women (Anders et al., 2020). The development of SLE has been attributed to a loss of self-tolerance, leading to the formation of autoantibodies to DNA and other nuclear antigens. Lupus nephritis (LN) may arise from the formation and deposition of immune complexes within the glomeruli of the kidneys. Affecting up to 60% of patients, LN is a major cause of morbidity and mortality in SLE. Clinically, LN is indicated by excessive proteinuria or decreased kidney function, which can progress to kidney failure requiring kidney replacement therapy (Chen et al., 2017). A kidney biopsy is currently the gold standard to confirm the diagnosis of LN and to establish histopathologic patterns as a guide for therapy. The kidney histology of LN depends on the site of accumulation of immunoglobulins, their antigen specificity, and their ability to activate complement and evoke an inflammatory response within the kidneys. Typically, LN is classified into six classes by the International Society of Nephrology/Renal Pathology Society (ISN/RPS) classification (Weening et al., 2004). Class III, IV, and V are active diseases, which require treatment with immunosuppressive agents to prevent kidney failure. Classes III and IV are characterized by endocapillary proliferation and deposition of immune complexes in the subendothelial space. Clinically, a patient is classified as class III if <50% of glomeruli from the biopsy section shows proliferation and is classified as class IV if ≥50% of glomeruli are involved. Because classes III and IV have rapid progression to kidney failure and are clinically treated with the same high dose of immunosuppressive drug regimen, they are practically considered as the same class. In class V, antibodies and complements induce cytotoxic injury on the podocytes (glomerular visceral epithelial cells), resulting in a non-exudative and non-proliferative capillary wall lesion with sub-epithelial immune deposits (membranous lesion). Membranous lesions may exist on their own (pure class V) or may co-exist with endothelial proliferation (mixed class III/IV + V). The prognosis of pure class V lesions is typically better than pure class III/IV (endothelial proliferation only), but moderate levels of immunosuppressive therapy may still be required. On the other hand, patients with mixed class III/IV + V may have reduced therapeutic response compared to patients with pure class III/IV (Najafi et al., 2001). The histopathological class of patients with LN is not static but may change during the course of therapy. It is technically difficult to classify proliferative LN (pure class III/IV) from membranous LN (pure class V) or mixed class III/IV + V clinically as both are involved with severe proteinuria and decreased kidney function. As a kidney biopsy is an invasive procedure and can lead to major complications, it is not appropriate for monitoring changes or multiple sampling. Thus, there is an urgent need for non-invasive biomarkers to diagnose and differentiate LN classes to timely determine the best course of treatment.

Metabolomics looks at changes in metabolites, which reflect alterations in phenotypes (Wishart et al., 2007; Wishart, 2019). Progress in metabolomics-based biomarker discovery depends on the advancement in analytical chemistry (Wishart, 2016) and bioinformatics tools (O'Shea and Misra, 2020; Cambiaghi et al., 2016; Grapov et al., 2018; Pomyen et al., 2020). Nuclear magnetic resonance (^1^H-NMR) spectroscopy and mass spectrometry (MS) are currently the most common techniques applied in metabolomics research (Wishart, 2016). Numerous metabolites have previously been reported to be associated with kidney diseases (Hocher and Adamski, 2017). Of these, tryptophan (Trp) and its metabolites in the kynurenine pathways (KP) have recently emerged as potential biomarkers for chronic kidney diseases (CKD) (Pawlak et al., 2010); however, their role in LN diagnosis is barely understood. Urine metabolites are non-invasive biomarkers that may provide a direct snapshot of pathophysiological processes within kidneys (Reyes-Thomas et al., 2011). In recent years, there have been few studies evaluating urinary metabolites for the classification of LN (Romick-Rosendale et al., 2011; Ganguly et al., 2020). Although these early studies provided valuable information, the studies were conducted in relatively small populations with semi-quantitative information (fold change), which impedes further comparisons of markers across studies. Furthermore, the use of these metabolites as potential clinical biomarkers relies on rigorous validation of data from larger cohorts as well as the quantitative measurement (absolute quantification) of those metabolites. Currently, how the pathogenesis of membranous lupus differs from other forms of LN is not fully known, and only a few biomarkers have been reported to separate membranous from proliferative LN (Almaani and Parikh, 2019; Ward and Bargman, 2016).

## Results

### Cohort characteristics and the measurements of urine protein-creatinine ratio and estimated glomerular filtration rate

**Figure 1:**
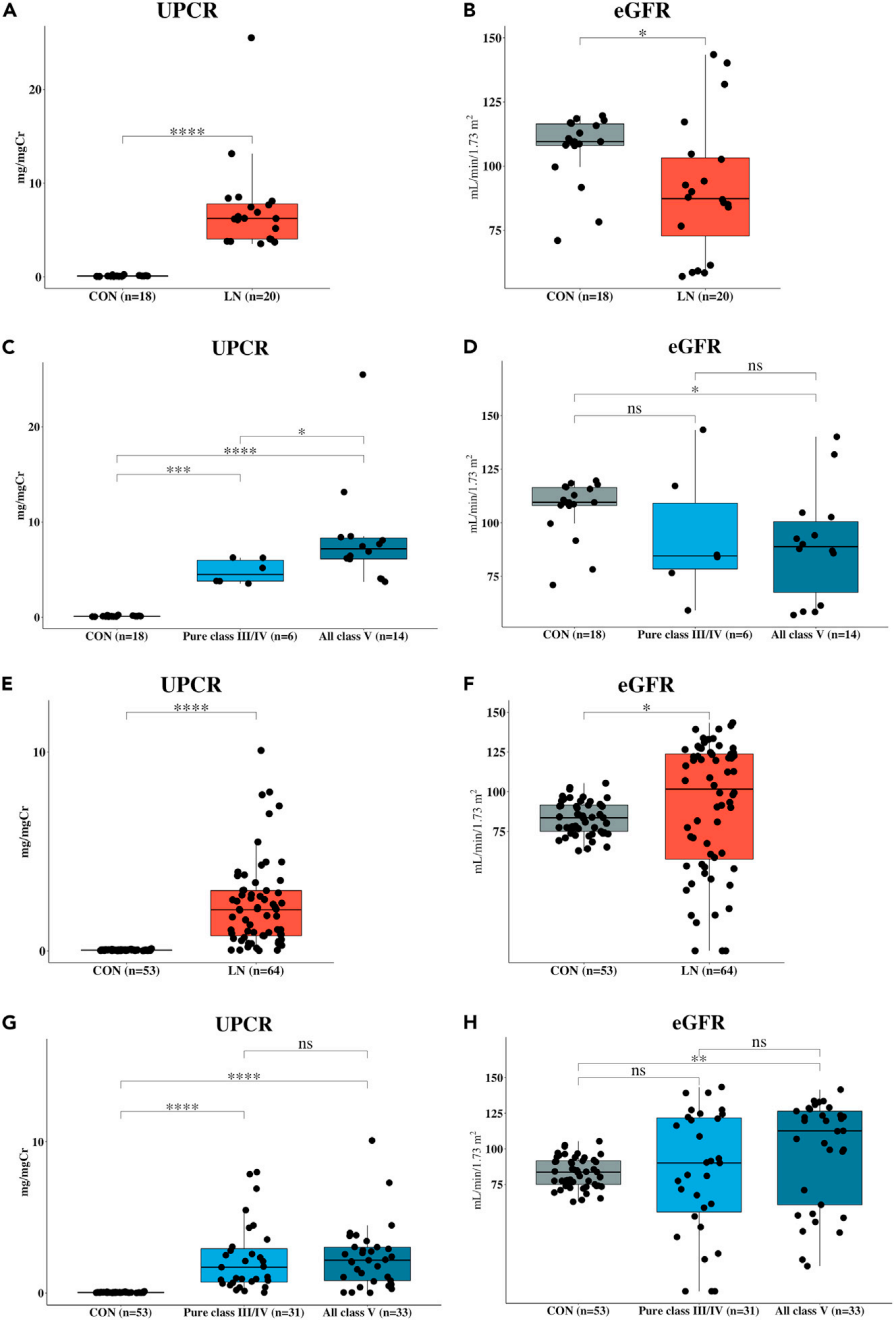
Box plots comparing levels of UPCR and eGFR between CON and LN, all class V and pure class III/IV in discovery cohort (A-D), and in the validation cohort (E-H) (A–H) Box plots comparing levels of UPCR and eGFR between CON and LN, all class V and pure class III/IV in discovery cohort (A–D), and in the validation cohort (E–H). Data are shown as ns (not significant) = p value > 0.05, ∗ = p value < 0.05, ∗∗ = p value < 0.01, ∗∗∗ = p value < 0.001, ∗∗∗∗ = p value < 0.0001, determined by Mann–Whitney U test.

The discovery cohort included 18 subjects from healthy controls (CON) and 20 patients from LN. Both groups had considerably more female than male subjects (Table 1). Proteinuria levels (urine protein creatinine ratio; UPCR) were significantly higher (p value < 0.0001) in LN compared to CON, whereas estimated glomerular filtration rate (eGFR) levels were significantly lower (p value < 0.05) in LN than CON (Figures 1A and 1B). In this cohort, LN classes comprised of pure class III/IV (N = 6), pure class V (N = 7), and mixed class III/IV + V (N = 7). To evaluate the effects of membranous components, we combined pure class V and mixed class III/IV + V (all class V; N = 14). Proteinuria levels were significantly different among the three groups (Figure 1C), whereas eGFR levels show only a significant difference between CON and all class V (Figure 1D).

**Table 1:** Demographic and clinical data of the study population

Characteristic	CON	LN
**Discovery cohort**		

Gender – No. (% in group)		
Male	5 (28)	2 (10)
Female	13 (72)	18 (90)
Age – Year		
Mean	44.3 ± 7.1	30.2 ± 10.3
Range	34–55	16–56
UPCR		
Mean	0.11 ± 0.06	7.25 ± 4.87
Range	0.06–0.25	3.54–25.49
eGFR – mL/min/1.73m^2^		
Mean	106.85 ± 13.65	90.87 ± 26.53
Range	71–119.7	57–143.4
LN class – No. (%)^a^		
Pure class III/IV	–	6 (30)
Mixed class III/IV + V	–	7 (35)
Pure class V	–	7 (35)

**Validation cohort**		

Gender – No. (% in group)		
Male	40 (75)	6 (9)
Female	13 (25)	58 (91)
Age – Year		
Mean	61.1 ± 4.1	32.3 ± 12.7
Range	55–69	15–65
UPCR		
Mean	0.04 ± 0.02	32.3 ± 12.73
Range	0.01–0.11	0.02–10.08
eGFR – mL/min/1.73m^2^		
Mean	83.37 ± 10.49	32.3 ± 12.73
Range	62.89–105.38	17.7–143.4
LN class – No. (%)^a^		
Pure class III/IV	–	31 (48.44)
Mixed class III/IV + V	–	18 (28.12)
Pure class V	–	15 (23.44)

UPCR, Urine protein creatinine ratio; eGFR, estimated glomerular filtration rate.

a LN class based on ISN/RPS classification by renal-biopsy proven.

The validation cohort included 53 subjects from CON and 64 patients from patients with LN. Of this, male subjects account for 9% and 75% of the LN and CON, respectively (Table 1). There were more male subjects in the CON group, whereas more than 90% were female in the LN group. In general, proteinuria levels were significantly higher (p value < 0.0001) in LN compared with the CON group (Figure 1E), similar to the eGFR levels (p value < 0.05) in the LN compared with CON (Figure 1F). For the LN class comparison, the proteinuria levels of pure class III/IV (N = 31) and all class V (N = 33) were significantly greater (p value < 0.0001) than that of CON (Figure 1G). The eGFR levels show only a significant difference between all classes V and CON (Figure 1H).

### Fum, Ket, Pyr, and Mal are the most significant metabolites separating lupus nephritis from controls

**Figure 2:**
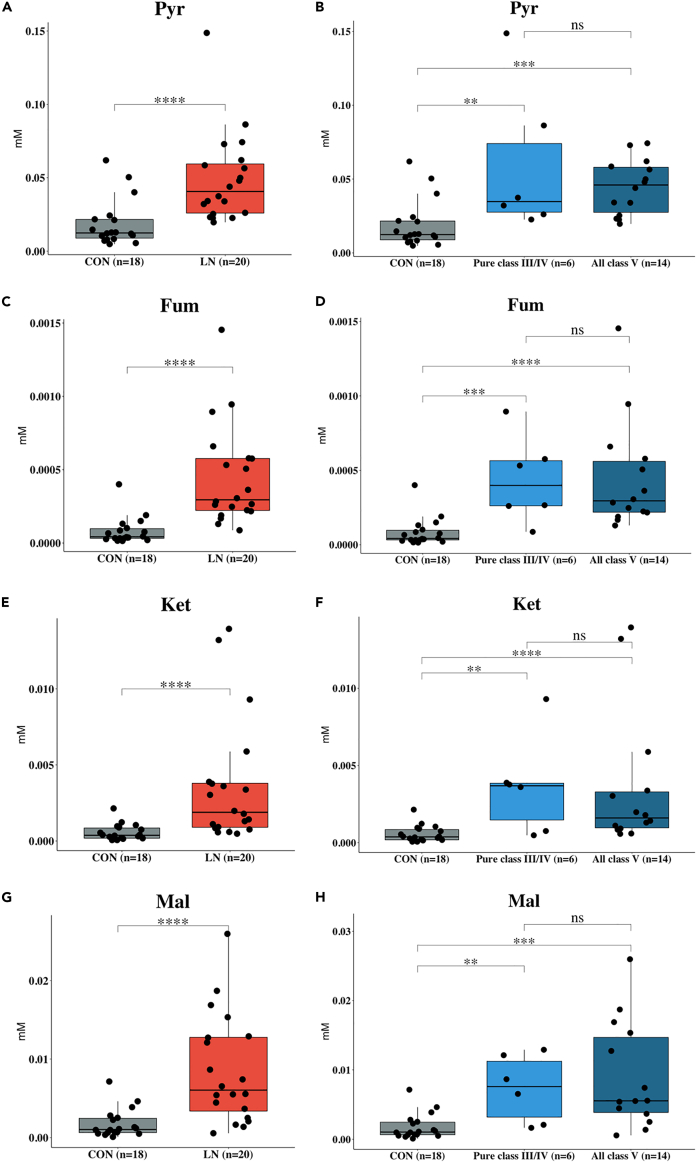
Box plots comparing concentrations of candidate metabolites between CON and LN, pure class III/IV and all class V in the discovery cohort (A–H) (A and B) pyruvic acid (Pyr), (C and D) fumaric acid (Fum), (E and F) α-ketoglutaric acid (Ket), and (G and H) malic acid (Mal). Data are shown as ns (not significant) = p value > 0.05), ∗ = p value < 0.05, ∗∗ = p value < 0.01, ∗∗∗ = p value < 0.001, ∗∗∗∗ = p value < 0.0001, determined by Mann–Whitney U test.

^1^H-NMR metabolite profiling in the discovery cohort resulted in a total of 43 metabolites. Of which, 36 compounds were identified using HMDB and an in-house database. The principal component analysis (PCA) score plot shows a clear separation between LN and CON groups, whereas no clear separation between all class V and pure class III/V (See also Figure S1A) was observed. The top 10 candidate metabolites were ranked by FDR-adjusted p value and fold change cutoff at 1.5 are present in Table S1. Under these criteria, pyruvic acid (Pyr) was found to be the most statistically significant metabolite separating LN from CON (p value < 0.0001). However, Pyr levels were not significant differences between pure class III/IV and all class V (See also Figure S2). Further results from the validation experiments by GC-MS/MS and LC-MS/MS showed that fumaric acid (Fum), α-ketoglutaric acid (Ket), Pyr, and malic acid (Mal) were statistically significant metabolites that can be used to discriminate CON from LN, but not among the three LN groups (Figure 2; Table S2).

### [Pic/Trp] ratio separates CON from LN; and pure class III/IV from all class V

The LC-MS/MS targeted analysis (absolute quantification in mM) of 10 metabolites in the KP was first performed on samples from the discovery cohort. The level of 3-hydroxykynurenine (3OH-Kyn) was significantly higher (p value < 0.05), while the Pic level was significantly lower (p value < 0.01) in LN than CON (see also Figures S3A, S3B, S3E, and S3F). In addition, urinary quinolinic acid (Qui) was found to significantly increased in patients with LN, albeit only in the validation cohort (See also Figure S3G). In the combined datasets, the levels of 3-hydroxyanthranilic acid (3OH-Ant) were also significantly different between LN and CON (see also Figure S3L). However, none of the individual targeted metabolites were able to distinguish pure class III/IV from all class V.

**Figure 3:**
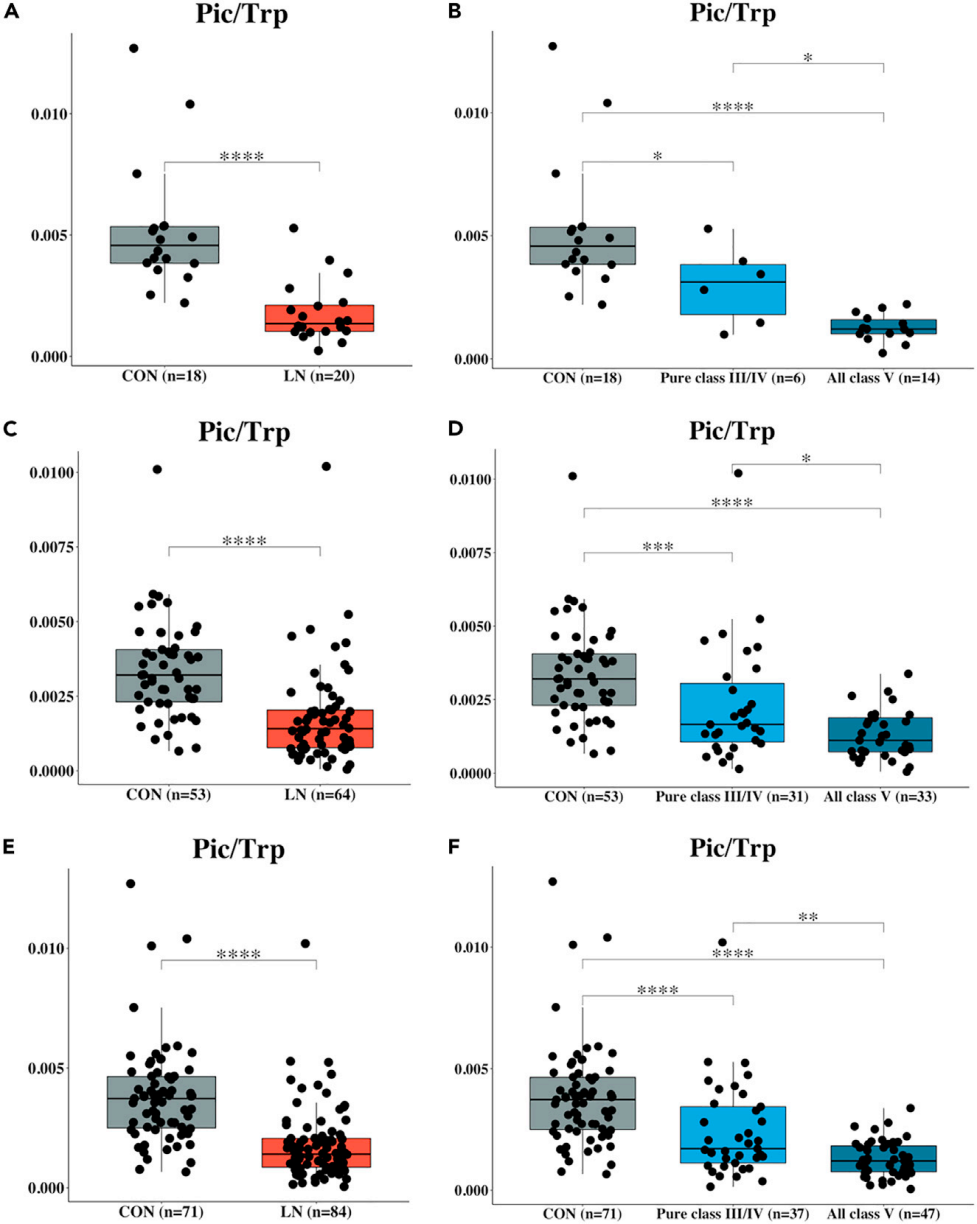
Box plot comparing the ratio of the final product (picolinic acid; Pic) and substrate (tryptophan; Trp) in the kynurenine pathway (KP) from the discovery cohort (A and B), the validation cohort (C and D), and combined cohort (E and F) (A–F) Box plot comparing the ratio of the final product (picolinic acid; Pic) and substrate (tryptophan; Trp) in the kynurenine pathway (KP) from the discovery cohort (A and B), the validation cohort (C and D), and combined cohort (E and F). The ratio of [Pic/Trp] shows a potential use for both LN diagnostics and membranous LN prediction. Data are shown as ns (not significant) = p value > 0.05, ∗ = p value < 0.05, ∗∗ = p value < 0.01, ∗∗∗ = p value < 0.001, and ∗∗∗∗ = p value < 0.0001, determined by Mann–Whitney U test.

However, we found that [Pic/Trp] ratio was able to separate healthy CON from patients with LN (p value < 0.0001) as well as pure class III/IV from all class V (p value < 0.05) in both the discovery cohort (Figures 3A and 3B) and the validation cohort (Figures 3C and 3D) and also in the combined datasets (Figures 3E and 3F).

### The combination of [picolinic acid/tryptophan], estimated glomerular filtration rate, and urine protein–creatinine ratio increases lupus nephritis classification performance

**Figure 4:**
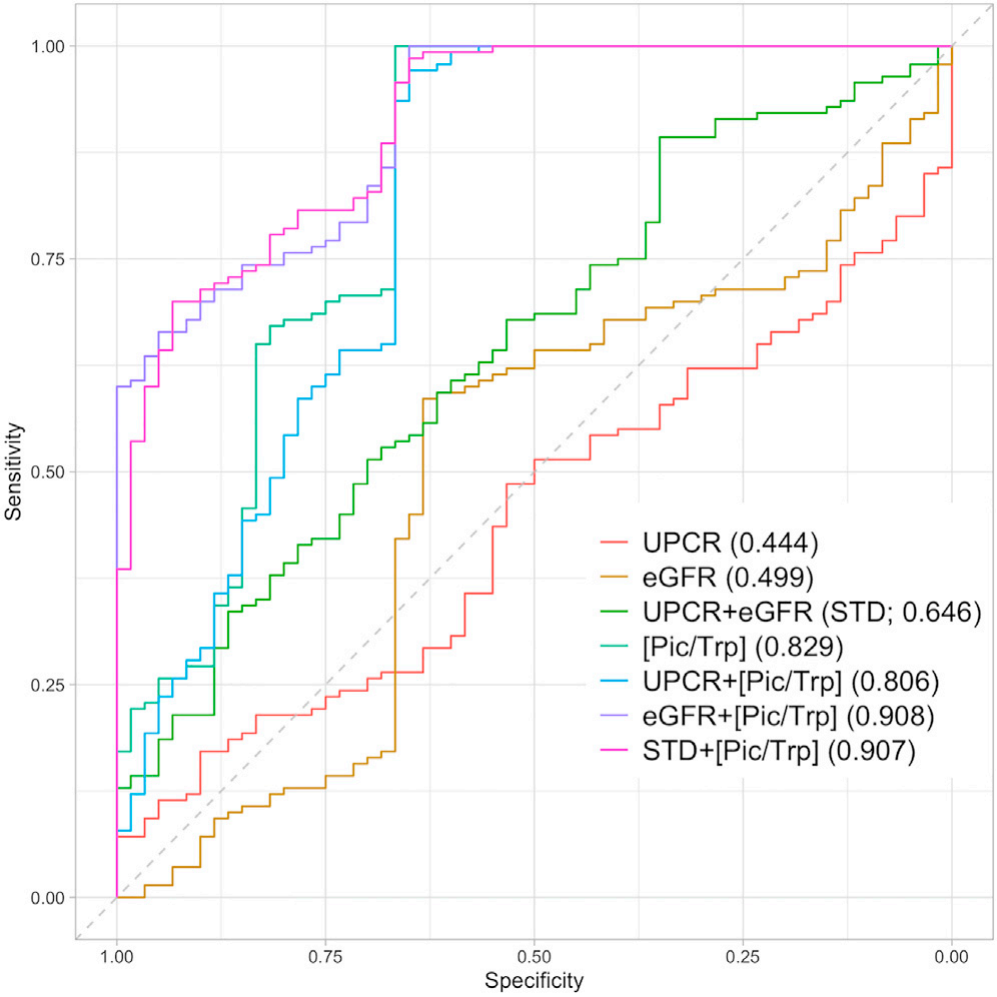
External validation of LN classification. ROC curves for discrimination between all membranous (all class V) and pure class III/IV Data are represented as parameters (AUC scores).

The predictability of [Pic/Trp] ratio, eGFR, and UPCR was assessed. We generated seven different LN classification models from two clinical parameters (eGFR and UPCR) and the [Pic/Trp] ratio (Figure 4). The area under curve (AUC) of [Pic/Trp] ratio was 0.829, which was higher than the AUC of eGFR (AUC = 0.499), UPCR (AUC = 0.444), and eGFR with UPCR (AUC = 0.646). The AUC of the model combining [Pic/Trp] ratio, eGFR, and UPCR was the highest (AUC = 0.91). Meanwhile, combinations of a clinical parameter, either eGFR or UPCR, with [Pic/Trp] ratio in the models yielded better predictive performance than using one clinical parameter alone. These results indicated that the use of the [Pic/Trp] ratio together with both eGFR and UPCR or the [Pic/Trp] ratio together with eGFR could significantly improve the LN classification of pure class III/IV from all class V.

### [Picolinic acid/tryptophan] ratio as a potential marker for membranous lupus nephritis

**Figure 5:**
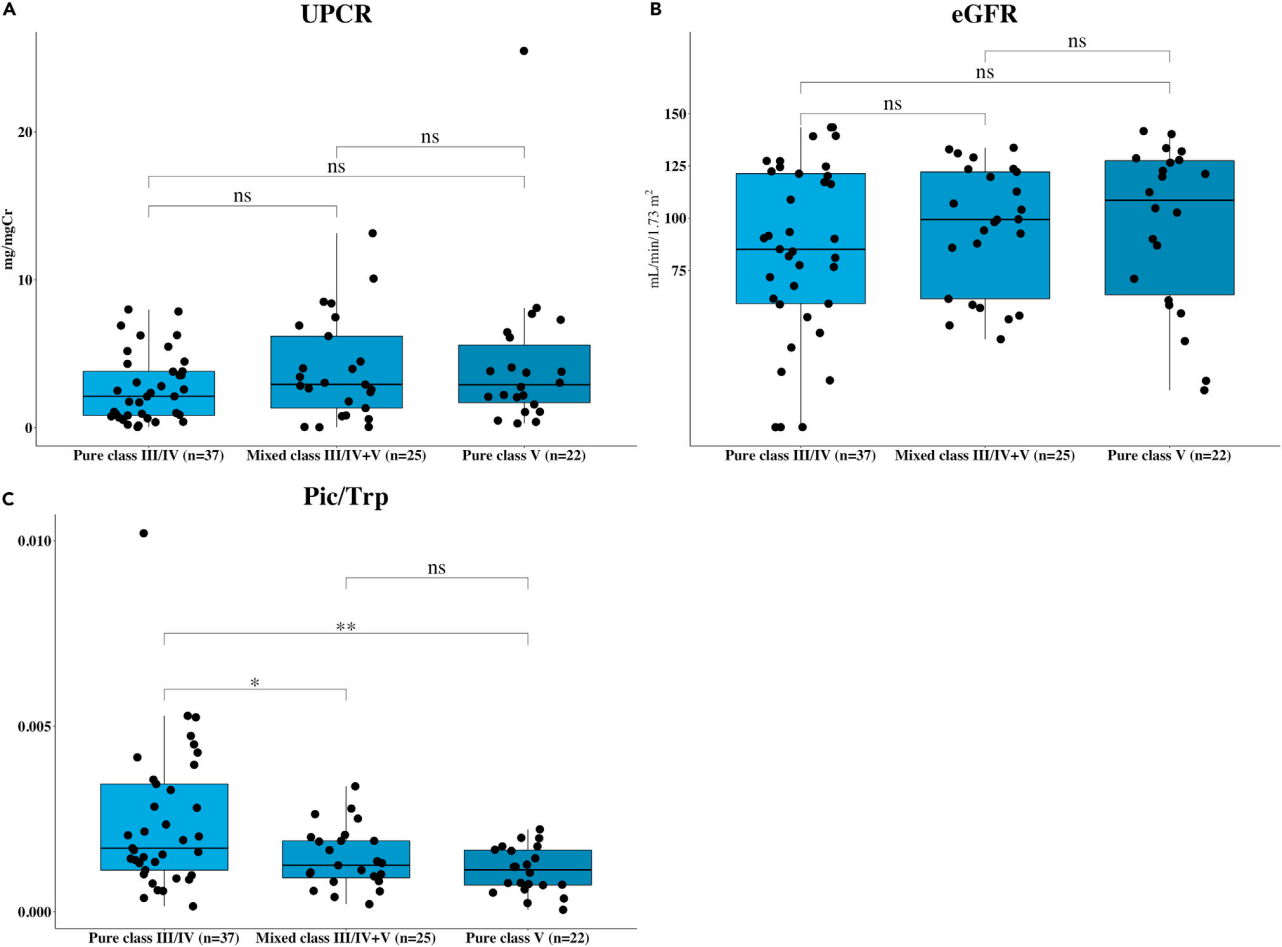
Box plots comparing levels of conventional biomarkers as UPCR (A) and eGFR (B), and potential biomarker [Pic/Trp] (C) among pure class III/IV, mixed class III/IV+V, and pure class V in the combined cohort (A–C) Box plots comparing levels of conventional biomarkers as UPCR (A) and eGFR (B), and potential biomarker [Pic/Trp] (C) among pure class III/IV, mixed class III/IV + V, and pure class V in the combined cohort. Data are shown as ns (not significant) = p value > 0.05, ∗ = p value < 0.05, ∗∗ = p value < 0.01, ∗∗∗ = p value < 0.001, and ∗∗∗∗ = p value < 0.0001, determined by Mann–Whitney U test.

We further compared the differences of eGFR, UPCR, and [Pic/Trp] ratio among the LN classes using the combined datasets. In total, the combined datasets consist of pure class III/IV (N = 37), pure class V (N = 22), and mixed class III/IV + V (N = 25**)**. There were no significant differences in proteinuria or eGFR among these three groups (Figures 5A and 5B). The [Pic/Trp] ratio was significantly higher in pure class III/IV compared to mixed class III/IV + V (p value = 0.0362) and pure class V (p value = 0.0099); however, there was no difference between pure class V and mixed class III/IV + V (Figure 5C). [Pic/Trp] ratios are not correlated with proteinuria levels or with eGFR in pure class III/IV, mixed class III/IV + V, and pure class V (see also Figures S4A–S4C).

## Discussion

LN is a form of glomerulonephritis and constitutes one of the most severe kidney-related manifestations of the autoimmune disease known as SLE. The treatment of patients with LN relies on the disease severity assessment, which requires renal biopsies. Thus, there is an apparent need for non-invasive biomarkers that can differentiate LN classes, proliferative, and membranous, which will greatly assist the treatment decisions. A few studies have explored urinary metabolites in patients with LN (Ganguly et al., 2020; Almaani and Parikh, 2019; Ward and Bargman, 2016). Nevertheless, a promising biomarker has not yet been reported. In this study, we aimed to discover urinary metabolite biomarkers that can differentiate CON from patients with LN and also differentiate pure proliferative LN (pure class III/IV) from membranous LN (all class V). Initially, we analyzed UPCR and eGFR data from both discovery and validation cohorts and found that both markers effectively separated LN from CON (Figures 1A, 1B, 1E, and 1F). However, both UPCR and eGFR were not consistently sensitive enough to separate pure class III/IV from pure class V in validation cohorts (Figures 1G and 1H). Thus, we concluded here that both UPCR and eGFR were molecular biomarkers that are only effective in separating patients with LN from healthy CON, but not sensitive enough for LN classification.

Next, we conducted multi-platform metabolomics analyses, which included metabolite profiling and targeted analysis. With the discovery cohort, the ^1^H-NMR metabolite profiling was able to detect 43 metabolites of which 36 were identified. Using PCA, the ^1^H-NMR metabolome result showed a clear class separation (See also Figure S1A) between all class V from CON and a little separation between all class V and pure class III/IV. These results suggest that concentrations of these metabolites, indeed, reflect different states of patients with LN. Among 10 candidate metabolites determined from the NMR dataset, Pyr was the most statistically significant metabolite for separating LN from CON. However, no metabolite was able to separate class III/IV from all class V. The enrichment analysis of these metabolites showed numerous metabolic pathways that are involved in LN, such as glycolysis, leucine degradation, TCA cycle, etc. However, it is noteworthy that these metabolites were annotated through matching with the HMDB library without validation with authentic standards. The identity of these metabolites needs to be confirmed before subsequent biological interpretation. Thus, we further analyzed these samples using GC-MS/MS quantitative in combination with the silylation derivatization method (Khoomrung et al., 2015) to confirm Pyr identity as well as other significant candidate metabolites previously identified in the ^1^H-NMR experiment. Owing to the GC-MS/MS sensitivity issue, hippuric acid (Hip), Mal, and succinic acid (Suc) were re-quantified using LC-MS/MS. Results from the targeted analyses using GC-MS/MS and LC-MS/MS (see also Table S2) revealed significant alteration of four metabolites, including Pyr, Fum, Ket, and Mal, in glycolysis and TCA cycle in patients with LN compared with CON (Figure 2). Nonetheless, none of the quantified metabolites were able to distinguish pure class III/IV from all class V on their own (Figure 2).

**Figure 6:**
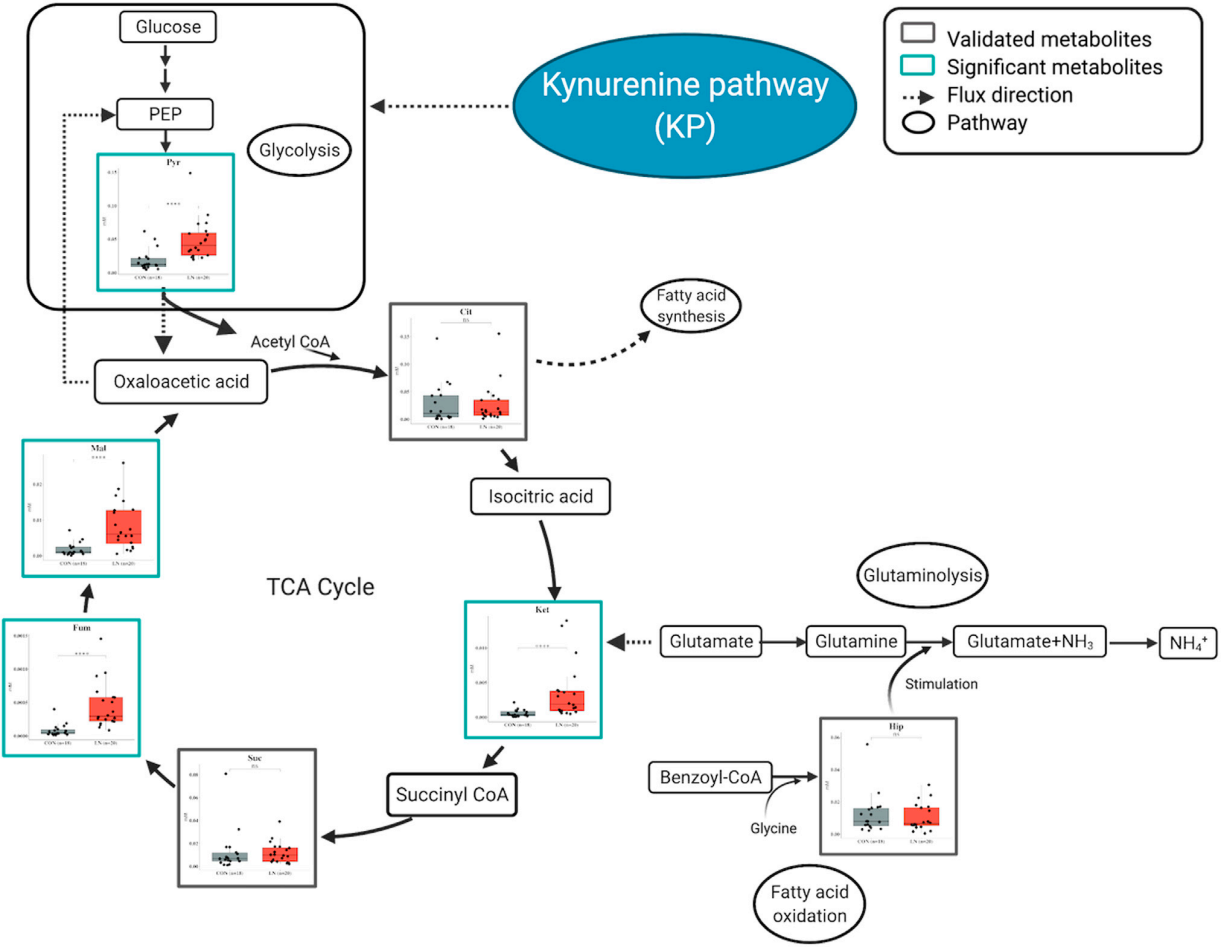
Associated metabolic pathways of candidate metabolites in the progression of LN involved in kynurenine pathway, glycolysis, and TCA cycle Data are shown as ns (not significant) = p value > 0.05, ∗ = p value < 0.05, ∗∗ = p value < 0.01, ∗∗∗ = p value < 0.001, and ∗∗∗∗ = p value < 0.0001), determined by Mann–Whitney U test. Figure was created with BioRender.com.

Increased urinary Pyr and TCA intermediate metabolites such as Ket, Fum, and Mal (Figure 6) may reflect increased T lymphocytes activation in LN. T lymphocytes in lymphoid organs and those infiltrating the kidneys have an essential role in the pathogenesis of SLE and LN by initiating and amplifying the inflammatory process (Sharabi and Tsokos, 2020). T cells metabolic pathways along with the presence of nutrients, that is, glucose, glutamine, and fatty acids, influence both the activation and function of immunomodulatory molecules, that is, cytokines, chemokines, and growth factors (Sharabi and Tsokos, 2020). The engagement of the T cell receptor by autoantigens, consequence in shifting of T cells from quiescence to activation, proliferation, and acquire effector functions. During the process, glucose is metabolized by both glycolysis and oxidative phosphorylation to generate energy. In glycolysis, glucose is first converted to Pyr and then to acetyl coenzyme A (acetyl-CoA) before entering the TCA cycle and finally resulted in adenosine triphosphate (ATP) products through the oxidative phosphorylation reaction (Gaber et al., 2019). In addition to glucose metabolism, immune cells can utilize metabolites in the TCA cycle to generate ATP by metabolizing glutamine via glutaminolysis *via* ATP to produce glutamate, which is subsequently metabolized to Ket. However, no significant difference in citric acid (Cit), Suc, and Hip levels was observed in the present study indicating the possibility of an alternate mechanism during LN progression.

**Figure 7:**
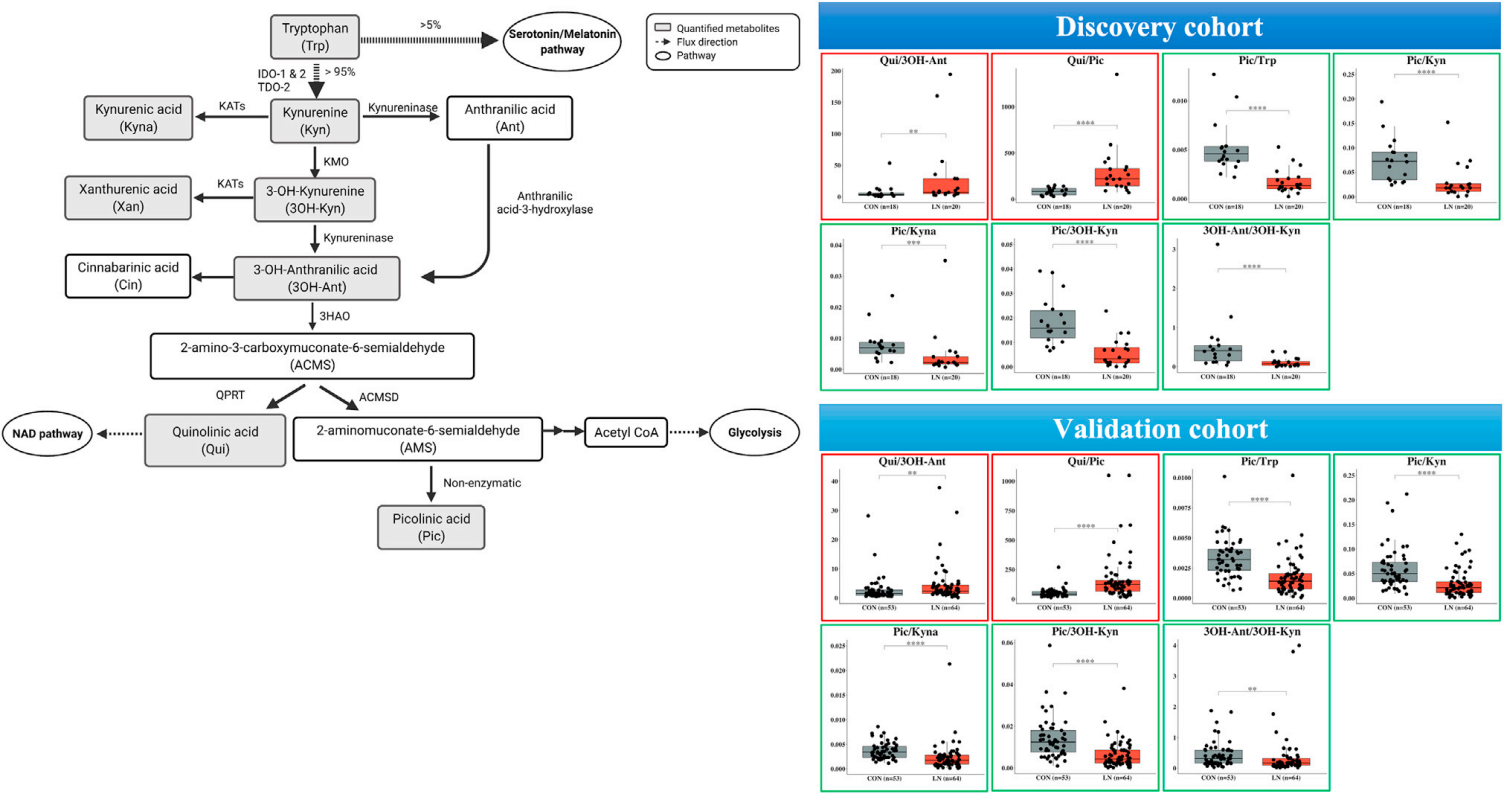
A metabolic pathway of metabolites in the kynurenine pathway (Savitz, 2020; Tan and Guillemin, 2019) Trp is predominantly converted into Kyn by the indoleamine 2,3-dioxygenases (IDO) and tryptophan 2,3-dioxygenase (TDO). Kyn can be further metabolized into Kyn by the kynurenine amino transferase isozymes (KATs). Kyn may be converted into Ant by kynureninase or 3OH-Kyn by kynurenine monooxygenase (KMO). 3OH-Kyn is converted into 3OH-Ant by kynureninase and metabolisms down the latter pathway to 2-amino-3-carboxymuconate-6-semialdehyde (ACMS) by the 3-hydroxyanthranilic acid oxygenase (3HAO) enzyme. ACMS is spontaneously degraded to form neurotoxic Qui, and can also be converted to 2-aminomuconate-6-semialdehyde (AMS) by the 2-amino-3-carboxymuconate-6-semialdehyde decarboxylase (ACMSD) enzyme. AMS rapidly cyclizes nonenzymatically to a neuroprotective Pic and shunts to acetyl-CoA synthesis to glycolysis. Qui is the endogenous source of nicotinamide and nicotinamide adenine dinucleotide (NAD+). Metabolites in this pathway and the ratio of the product and substrate were compared among subject groups (Red square represents up-regulation and green square represents down-regulation). More information is shown in Table S3. The Figure was created by BioRender.com.

In addition to the ^1^H-NMR experiment and its validations in the discovery cohort, we performed a targeted analysis of 10 metabolites in KP, including anthranilic acid (Ant), cinnabarinic acid (Cin), kynurenic acid (Kyna), kynurenine (Kyn), Pic, Qui, Trp, xanthurenic acid (Xan), 3OH-Ant, and 3-hydroxykynurenine (3OH-Kyn). Although several metabolites in KP have been linked with CKD (Pawlak et al., 2010), their involvement in LN has not yet been reported. Typically, KP is the major metabolic pathway of Trp degradation, ∼95% from dietary Trp disposal in the liver (Badawy, 2017) (Figure 7), generating molecules that are involved in many physiological and pathological processes including redox homeostasis, gluconeogenesis inflammation, carcinogenesis, and apoptosis (Badawy, 2017). KP is known to be upregulated as part of an activated immune response (Chen and Guillemin, 2009) and catalyzed by two key enzymes: tryptophan 2,3-dioxygenase (TDO) and indoleamine 2,3-dioxygenase 1 (IDO1). IDO present in immune cells is induced by interferon-gamma (IFN-γ), which is activated in SLE. Higher blood levels of Kyn and lower blood levels of Trp have been observed in patients with SLE (Xiang et al., 2010, Widner et al., 2000; Pertovaara et al., 2007; Åkesson et al., 2018). Increased levels of blood [Kyn/Trp] ratio is consistent with increased IDO activity, which is often correlated with disease activity (Badawy and Guillemin, 2019), but kidney involvement has not yet been explored in earlier studies. Additionally, there is currently a lack of information on urinary kynurenine pathway metabolites (KPMs) in patients with LN. Our LC-MS/MS target analysis of urine (see also Figure S3) revealed significant alteration of 3OH-Kyn, Pic, Qui, and 3OH-Ant in patients with LN compared to healthy CON. The level of urinary 3OH-Kyn significantly increased, whereas Pic was significantly decreased in patients with LN compared to CON in both cohorts. On the other hand, Qui significantly increased only in the validation cohort. Variations of individual urinary KP metabolites in LN may reflect altered synthetic or degradation pathways in the systemic circulation or within the kidneys as well as altered infiltration or tubular transport. Therefore, the ratios of KPMs were assessed. Increased ratios of [Qui/Pic] and [Qui/3OH-Ant] in LN in both the discovery and validation cohort imply that pathways related to the formations of 3OH-Kyn and downstream metabolites such as Qui were preferentially up-regulated (Figure 7; Table S3). In contrast, consistent decreases in [Pic/Trp], [Pic/Kyn], [Pic/Kyna], [Pic/3OH-Kyn], [Pic/Xan], [Pic/3OH-Ant], and [3OH-Ant/3OH-Kyn] in LN (See also Table S3) suggest specific decreases in Pic synthetic pathway or possibly rapid shunting to acetyl-CoA synthesis to glycolysis. Metabolites in KP may contribute to the pathogenesis of LN by modifying immune responses or by directly affecting kidney cell function. KPMs activate the mTOR signaling pathway, suppress T effector cell proliferation, and promote the development of regulatory T cells (Sharabi and Tsokos, 2020). Targeted deletion of the enzyme kynurenine 3-monooxygenase (KMO) in mice leads to proteinuria accompanied by the upregulation of Kyn, Kyna, and reduced Ant and presumably 3OH-Kyn and its downstream metabolite (Korstanje et al., 2016). Glomerular expression of KPMs was also reduced in proteinuric diabetic kidney disease, suggesting that alterations in local KP metabolism may have a direct role in the pathogenesis of glomerular diseases (Mor et al., 2020). The roles of specific KPMs may differ and have not been fully characterized. 3OH-Kyn was the most potent inducer of reactive oxygen species generation, mitochondria function impairment, and apoptosis in neurons (Okuda et al., 1998) and was found to produce suppression of mesangial proliferation (Yoshimura et al., 2009). In contrast, Qui may increase mesangial cell proliferation and collagen synthesis. There is limited information on the role of Pic, which is an endogenous neuroprotectant, a natural iron and zinc chelator, which can suppress the proliferation of CD4+ T cells (Chen and Guillemin, 2009; Prodinger et al., 2016). The highest activity of human 2-amino-3-carboxymuconate- 6-semialdehyde decarboxylase (ACMSD) or picolinate carboxylase is in the kidney suggesting that the decrease in kidney Pic synthesis may contribute to lower urinary Pic excretion in LN (Badawy, 2017). A low level of Pic, in turn, may enhance cellular proliferation as Pic has a suppressive function on normal rat kidney cells (Fernandez-Pol et al., 1977). However, most studies about KPMs in patients with CKD aimed at the identification of plasma metabolites or focus on diabetic nephropathy (DN) (Badawy and Guillemin, 2019; Rhee et al., 2013; Tan and Guillemin, 2019; Hirayama et al., 2012; Zhao, 2013). This study is the first study proposing the potential metabolic abnormalities occurring in the KP of patients with LN.

In the perspective of the LN biomarker from KP metabolomics analysis, our results from the discovery and validation cohorts show that the ratio of [Pic/Trp] was both specific and sensitive for separating membranous LN from pure proliferative LN. Furthermore, the analysis of the combined datasets provides stronger evidence (decreased of the corrected p value from 0.05 to 0.001) of [Pic/Trp] ratio predictive performance (Figures 3E and 3F). The use of the [Pic/Trp] ratio to discriminate membranous LN is also a complement to clinical parameters (Figures 3A and 3B). Typically, the quality of the data (data normalization) is one of the biggest concerns that might impact the accuracy of a biomarker, especially when identifying biomarkers from a urine sample. In this study, two approaches were employed to remove technical and biological variations during data processing (Wanichthanarak et al., 2019). In quantitative analysis of metabolites in KP by LC-MS/MS, the internal standard (IS) with known amounts was added to urine samples before sample preparation. The concentrations of the target analytes were then calculated by normalizing each target analyte peak area to the peak area of IS. Therefore, the addition of IS accounts for technical variations that would occur during sample extraction and instrumental analysis. Second, the urinary biomarker normalization in the context of renal biomarkers, parameters commonly used are urinary creatinine (UCr), urine volume (uVol), or urinary cystatin C (uCysC). In some cases, biomarkers were determined without normalization. The normalization with UCr is often recommended as a standard normalization method when compared to others (Adedeji et al., 2019), even though the UCr excretion can be variable across individuals and even within the same individual under nonsteady-state conditions (e.g., in acute kidney injury (AKI) and after kidney transplantation) (Waikar et al., 2010). Our study chose to normalize the metabolite concentrations by UCr. The normalization of individual biomarkers was calculated by dividing the concentration of metabolites (in mM) by the respective Cr concentration (UCr; mM). The equation is described as follows.

$$\text{Normalized}\;{\text{metabolites}}_{i}=\frac{\left[{\text{Metabolites}}_{i}\right]}{\left[{\text{UCr}}_{i}\right]}$$

where [Metabolites_*i*_] = concentration of metabolites (mM) in subject *i.*

[UCr_*i*_] = concentration of urine creatinine (mM) in subject *i.*

In the case of [Pic/Trp] ratio, the normalized [Pic/Trp] ratio is calculated as follows:

$$\text{Normalized}\left[\frac{\text{Pic}}{\text{Trp}}\right]\;\text{ratio}=\frac{\text{Normalized}\;\left[\text{Pic}\right]}{\text{Normalized}\;\left[\text{Trp}\right]}=\left[\frac{\text{Pic}}{\text{UCr}}\right]/\left[\frac{\text{Trp}}{\text{UCr}}\right]=\left[\frac{\text{Pic}}{\text{Trp}}\right]$$

From the calculation above, the [Pic/Trp] ratio is, in other words, equal to unnormalized [Pic] divided by unnormalized [Trp]. Thus, the analysis of the [Pic/Trp] ratio discovered in this study is not impacted by the choice of the normalization method.

The mechanisms behind the lower urinary [Pic/Trp] in membranous lupus nephropathy are currently unknown. As the urinary [Pic/Trp] levels were similar between pure class V and mixed class III/IV + V and did not correlate with proteinuria and eGFR (Figures 5A–5C), we hypothesize that decreased [Pic/Trp] was specific to the membranous lesion and podocyte injury. Although the exact site of Pic synthesis within the kidney is not fully known, the enzyme KMO responsible for the synthesis of the upstream metabolite, 3OH-Kyn, has been located at the podocytes as well other glomerular cells. This is indicative of local synthesis (Korstanje et al., 2016). The fact that glomerular podocytes are highly differentiated cells with limited capacity to regenerate may limit the ability to compensate when damaged by the complement components as part of the pathogenesis of class V lesions (Cellesi et al., 2015).

To our knowledge, this study is the first to investigate the urinary excretion of KPMs in LN and with the largest number of subjects. We implemented multi-platform metabolomics to identify and quantitate urinary excreted metabolites in patients with LN**.** We identified the [Pic/Trp] ratio as a good biomarker for the identification of membranous lesions in LN and later validated its performance in an independent cohort. Up until now, there has been no sensitive biomarker for this condition. In the future, the [Pic/Trp] ratio could be combined with other biomarkers that are selective for LN class III/IV to enable the non-invasive monitoring of LN progression. The discovery of low urinary Pic in LN should stimulate further research investigating the role of Pic in LN and other kidney diseases. We hypothesize that Pic may be directly responsible for kidney pathology, which may potentially lead to novel therapy to restore Pic levels in patients with LN.

The urinary [Pic/Trp] ratio has the potential to be used as a biomarker that is highly specific for membranous lesions in LN. Additional studies with long-term follow-up periods in different populations are necessary to confirm the universal application of this biomarker. Current findings may contribute to the development of an alternative non-invasive yet reliable and sensitive for early diagnosis of LN using the metabolomics-based methodology.

### Limitations of the study

This study has several limitations. First, urine samples could be collected only at the time of renal biopsy. Follow-up specimen collection would provide further insight into the benefit of the [Pic/Trp] ratio for monitoring the disease progression and treatment outcomes. Second, this study determined UPCR using spot urine sampling instead of 24-h urine sampling, which is the standard practice. However, 24-h urine collections are difficult to perform, and spot UPCR provides a reasonable estimate in clinical practice (Fanouriakis et al., 2020).

## STAR★Methods

### Key resources table

**Table:** 

REAGENT or RESOURCE	SOURCE	IDENTIFIER
**Chemicals, Peptides, and Recombinant Proteins**		

Acetonitrile, Optima™ LC/MS Grade	Fisher Chemical™	CAS #: A955-4
Methanol, Optima™ LC/MS Grade	Fisher Chemical™	CAS #: A456-4
Formic Acid, 99.0+%, Optima™ LC/MS Grade	Fisher Chemical™	CAS #: A117-50
Potassium dihydrogen phosphate	Merck	CAS #: 7778-77-0
Isopropanol, Optima™LC/MS Grade	Fisher Chemical™	CAS #: A461-212
Deuterium oxide	Merck	CAS #: 7789-20-0
Sodium azide	Merck	CAS #: 26628-22-8
3-(Trimethylsilyl) propionic acid-D_4_ sodium salt (TSP-D_4_)	Cambridge Isotope Laboratories, Inc	CAS #: DLM-48-1

**Deposited Data**		

Metabolomics data	Mendeley Data	https://doi.org/10.17632/srcz7scz67.2

**Software and Algorithms**		

MATLAB software (version R2014a)	MathWorks, Natrick, MA, USA	https://www.mathworks.com
Agilent MassHunter software (version 10.0)	Agilent Technologies, USA	https://www.agilent.com/en/promotions/masshunter-mass-spec
R (version 3.6.3)	R Core Team (2020)	https://cran.r-project.org

**Other**		

Mass spectrometer for LC	Waters^TM^	Xevo TQ-S
HSS T3 (130Å, 1.8 μm, 2.1 mm × 100 mm)	Waters^TM^	186003539
NMR	Bruker Avance III	400 MHz Bruker DRX spectrometer
HP-5MS column (30 m, 0.25 mm i.d., 0.25 u)	Agilent Technologies	AG19091S-433
Gas chromatography	Agilent Technologies	7890B GC System
Mass spectrometer for GC	Agilent Technologies	7000D Triple Quadrupole GC/MS
Auto Sampler System for GC	CTC Analytics AG	PAL Auto Sampler System
HSS T3 VanGuard (130Å, 1.8 μm, 2.1 mm x 5 mm)	Waters^TM^	186003976
Liquid chromatography	Waters^TM^	ACQUITY UPLC I-Class

### Resource availability

#### Lead contact

Further information and requests for resources, datasets, and protocols, should be directed to and will be fulfilled by the lead contact, Sakda Khoomrung (sakda.kho@mahidol.edu).

#### Materials availability

This study did not generate new unique reagents.

### Experimental model and subject details

#### Overview of the study

In this study, we first used a discovery cohort and applied untargeted NMR-based metabolomics analysis to identify candidate metabolites that can separate healthy controls (CON) from LN patients; and pure proliferative LN (class III/IV) from membranous LN (class V). The candidate metabolites identified from the ^1^H-NMR experiment were subsequently validated by GC-MS/MS and LC-MS/MS. Secondly, we searched for potential biomarkers by targeting previously speculated metabolites in KP in the discovery cohort. Finally, the potential candidate metabolites identified in the discovery cohort (from all experiments) were further validated in the validation cohort. Our objectives are to i) identify urinary metabolites that can differentiate LN patients from healthy controls and ii) identify urinary metabolites that can distinguish membranous LN from pure proliferative LN.

#### Subjects

LN patients and CON subjects were recruited from Ramathibodi Hospital, Bangkok, Thailand. Patients, who fulfilled at least four of the American College of Rheumatology 1982 revised criteria for SLE (Hochberg, 1997) and were referred to kidney biopsy for clinical indications of proteinuria ≥ 0.5g/24 hours (Fanouriakis et al., 2020) were included. Patients with drug-induced lupus, active malignancies, overlapping syndrome, urinary tract infection were excluded. Subjects in CON groups had a physical examination and routine laboratory tests, including urinalysis, serum creatinine, and fasting blood glucose. Subjects with chronic illnesses, including hypertension, diabetes, cancer, or kidney diseases, were excluded from the study. The study was approved by the Ethics Committee of the Faculty of Medicine, Ramathibodi Hospital, Mahidol University, Bangkok, Thailand, and conducted in compliance with the Helsinki Declaration. All participants gave written informed consent. The age and gender of subjects in individual cohorts were shown in Table 1. Both groups (CON and LN) and cohorts had considerably more female than male subjects. The age of subjects from both cohorts ranged from 15 to 69 years.

### Methods details

#### Kidney histopathology classification

Kidney tissues were fixed (Glyo-Fixx), Thermo Fisher Scientific, USA) and paraffin-embedded, and sections (2 μm) were processed for light microscopy (hematoxylin and eosin, periodic acid Schiff, Masson’s trichrome, and silver staining), immunofluorescence, and electron microscopy. A specialist nephropathologist, who did not have knowledge of the laboratory data, classified according to the ISN/RPS (Bajema et al., 2018). In this study, we classified LN patients into four groups. First, patients with class III or IV without class V components were classified as pure class III/IV (See also Figure S5B). Second, patients with class V without class III/IV features were classified as “pure class V” (See also Figure S5C). Third, patients with both III/IV and V components were classified as mixed class III/IV+V (See also Figure S5D). Fourth, to identify biomarkers for membranous histological features, patients with “mixed class III/IV+V” and “pure class V” were combined as “all class V”.

#### Blood and urine collection, and analysis

Both morning urine and blood samples were collected under sterile conditions within the same day of biopsy/recruitment. Urine samples were centrifuged at 3,500 rpm for 10 min at 4°C. The supernatant was then aliquoted and stored at −80°C until the analysis. Common blood biochemical parameters were measured by an ISO 15189 accredited laboratory. Serum creatinine (SCr) and urine creatinine (UCr) were measured by an enzymatic assay using a Dimension ExL analyzer (Siemens Healthcare Diagnostics, Newark, DE, USA). Urine protein (Uprot) was measured by a modified pyrogallol red-molybdate method. Uprot was reported as urine protein creatinine ratio (UPCR in mg/mgCr). The estimated glomerular filtration rate (eGFR in mL/min/1.73 m^2^) was calculated using the CKD-EPI equation (Levey et al., 2009):

$$\text{GFR}=141\times \min{\left(\text{SCr}/\kappa ,1\right)}^{\alpha }\times \max{\left(\text{SCr}/\kappa ,1\right)}^{-1.209}\times 0.993^{\text{Age}}\times 1.018\left[\text{if}\;\text{females}\right]\times 1.159\left[\text{if}\;\text{black}\right]$$

Where SCr = serum creatinine (mg/dL).

κ = 0.7 for females and 0.9 for males.

α = −0.329 for females and −0.411 for males.

min indicates the minimum of SCr/κ or 1.

max indicates the maximum of SCr/κ or 1.

#### Chemical, reagents, and standards

Potassium dihydrogen phosphate (KH_2_PO_4_), deuterium oxide (D_2_O), and sodium azide (NaN_3_) were purchased from Merck (Darmstadt, Germany). 3-(Trimethylsilyl) propionic acid-D_4_ sodium salt (TSP-D_4_), used as the internal standard reference for ^1^H-NMR analysis, was purchased from Cambridge Isotope Laboratories (Andover, MA, USA). High-performance LC (HPLC)-grade acetonitrile (ACN) and methanol (MeOH) were purchased from RCI Labscan (Bangkok, Thailand) and Fisher Chemical (Massachusetts, USA). Formic acid and isopropanol (IPA) were purchased from Fisher Chemical (Massachusetts, USA). HPLC quality water was purified using a Milli-Q water system from Millipore (Molsheim, France). All standards used in this study were of analytical grade (96–99% purity). The following standards: pyruvic acid (Pyr; 98%), citric acid (Cit; 98%), succinic acid (Suc; ≥98%), malic acid (Mal; ≥98%), lactic acid (Lac), oxalic acid (Oxa), oxaloacetic acid (OAA; ≥ 97%), hippuric acid (Hip; 98%), fumaric acid (Fum), salicylic acid-D_6_ (Sal-D_6_; 98%), 3-hydroxyanthranilic acid (3OH-Ant; 97%), picolinic acid (Pic; 99%), quinolinic acid (Qui; 99%), xanthurenic acid (Xan; 96%), cinnabarinic acid (Cin; 98%), malonic acid (Malo; > 99%), and methylmalonic acid (MMA; > 99%) were obtained from Sigma-Aldrich (Missouri, USA). Anthranilic acid C_13_ (Ant-C_13_; 99%) was purchased from Cambridge Isotope Laboratories (Massachusetts, USA). α-ketoglutaric acid (Ket; ≥98.5%) was purchased from Glentham (United Kingdom).

#### Metabolite profiling by ^1^H-NMR spectroscopy

##### Preparation of urine for NMR measurement

A 540 μL of a urine sample was mixed with 60 μL of D_2_O, 67 μL of NMR urine buffer containing 1.5 M KH_2_PO_4_, 2 mM NaN_3_, and 1% TSP-D_4_. The mixture was vortexed for 10 s and centrifuged at 12,000× g (4°C) for 15 min. A 600 μL of the supernatant was transferred to an NMR glass tube (Wilmad-LabGlass, Vineland, NJ, USA) before NMR analysis.

##### NMR measurement and data processing

The urine samples from the previous section were analyzed by a 400 MHz Bruker DRX spectrometer (Bruker Avance III, Bruker BioSpin, Germany) at a ^1^H frequency of 400 MHz with a temperature of 300 K. A standard one-dimensional (1D) NMR pulse sequence (recycle delay [RD]-90°-t1-90°-tm-90°- acquire free induction decay) was used with t1 fixed to 3 μs. The suppression of the water peak was achieved by selective irradiation during a recycle delay of 2 s and mixing time (TM) of 100 ms. The 90 degree-pulse was adjusted to approximately 10 μs. Experiments with Carr–Purcell–Meiboom–Gill (CPMG) pulse sequence was run with 64 scans in total and recorded into 72 K data points with a spectral width of 20 ppm. The urinary spectra were calibrated with the TSP-D_4_ peak (δ^1^H 0.00 ppm), phased, and baseline correction using TopSpin 3.0 (Bruker, Germany). The pre-processed spectra were imported into MATLAB software (MathWorks, R2014a, Natrick, MA, USA) and digitized with a resolution of 0.0005 ppm. Spectral regions containing the TSP-D_4_ peak (δ^1^H −1.00 to −0.005 ppm), water (δ^1^H 4.70–4.90 ppm), and urea (δ^1^H 5.71–6.00 ppm) were removed. Spectral data normalization was performed using the probabilistic quotient normalization method. For the peak alignment, recursive segment-wise peak alignment (RSPA) was applied to the remaining spectral data.

#### Quantification of metabolites in kynurenine pathway

The targeted analysis by LC-MS/MS of 10 metabolites in the KP included anthranilic acid (Ant), cinnabarinic acid (Cin), kynurenic acid (Kyna), kynurenine (Kyn), picolinic acid (Pic), quinolinic acid (Qui), tryptophan (Trp), xanthurenic acid (Xan), 3-hydroxyanthranilic acid (3OH-Ant), and 3-hydroxykynurenine (3OH-Kyn). The urine samples were prepared based on a previously published protocol (Zhu et al., 2019) with minor modifications. Briefly, a 50 μL of each urine sample was mixed with 200 μL of MeOH/ACN (1:1, v/v) containing 100 ng of anthranilic acid C_13_ (Ant-C_13_) as an internal standard (IS). The mixture was vortexed for 30 s and sonicated for 10 min (room temperature). The mixture was then left overnight (−20°C) for protein precipitation before centrifugation at 13,000 rpm (4°C) for 15 min. The supernatant was transferred to a new test tube and evaporated to dryness (room temperature) using a vacuum concentrator (Labconco, MO, USA). The dried sample was reconstituted in 100 μL of Milli-Q H_2_O (containing 0.1% formic acid), vortexed for 30 s, and sonicated (room temperature) for 10 min. The reconstituted sample was centrifuged at 13,000 rpm (4°C) for 15 min. The supernatant was kept at −80°C before analysis. The LC-MS/MS measurement conditions were slightly modified from the previously published protocol (Limjiasahapong et al., 2021). Briefly, the measurement was performed on a Waters Acquity I-Class UPLC coupled with a Xevo TQ-S MS/MS interfaced by an electrospray ionization source (ESI). The ESI was operated in the positive ion mode as; capillary voltage 1.5 kV; source temperature 150°C; desolvation gas (N_2_) at 550°C with a flow of 900 L/h; cone gas flow 150 L/h; nebulizer gas (Ar) 7.0 bar; collision gas 0.25 mL/min. The tandem mass spectrometry (MS/MS) was operated in the multiple reaction monitoring (MRM) mode. N_2_ and Ar were used as desolvation and collision gases, respectively. MRM transitions for all the target analytes are given in Table S4. A volume of 5 μL of a standard or sample was injected onto an HSS T3 column, 2.1 × 100 mm, 1.8 μM column (Waters, Milford, MA, USA) at 30°C with a constant flow rate of 0.3 mL/min. Mobile phases consisted of (A) 0.1% formic acid in Milli-Q water and (B) 0.1% formic acid in ACN. The gradient program started at 99% A, decreased to 70% over 7.0 min, then decreased to 30% at 9 min, and return to the initial condition at 10 min and held for 4.0 min. The total runtime was 14 min. The weak and strong needles wash solutions were 5% ACN and H_2_O/ACN/MeOH/IPA (1:1:1:1), respectively. Quantitative analysis (absolute quantification) was achieved by external calibration curves of each standard prepared in pooled urine samples (standard addition) (Limjiasahapong et al., 2021). The linear ranges of the calibration standards are as follows: 3.5–1,000 ng/mL for Ant; 4.5–80 ng/mL for Cin; 8–2,500 ng/ml for Kyna; 8–5,000 ng/ml for Kyn; 1.4–400 ng/ml for Pic; 40–5000 ng/ml for Qui; 7–2000 ng/ml for Trp; 2–200 ng/ml for Xan; 900–14,000 ng/ml for 3OH-Kyn; 300–2,500 ng/ml for 3OH-Ant and 8–2,000 ng/ml for Ant-C_13_.

#### LC-MS/MS analysis of urinary metabolites

Urine extraction was carried out based on the published protocol by Zhu et al., (2019). Briefly, 5 μL of 0.1 nmol salicylic acid-D_6_ (Sal-D_6_), used as an IS, was spiked into 95 μL of a urine sample followed by 400 μL of MeOH/ACN (1:1, v/v). The mixture was vortexed for 30 s, sonicated for 10 min (room temperature), and left overnight at −20°C for protein precipitation. The mixture was then centrifuged at 13,000 rpm (4°C) for 15 min, transferred to a new tube, and evaporated to dryness using a vacuum concentrator (Labconco, MO, USA). The dried sample was reconstituted in 100 μL of Milli-Q water containing 0.1% formic acid, vortexed for 30 s, and sonicated (room temperature) for 10 min. The sample was centrifuged at 13,000 rpm (4°C) for 15 min. The measurement was performed using the Waters Acquity I-Class UPLC coupled with a Xevo TQ-S MS/MS as previously described. The ESI was operated in the negative ion mode. The quantification of Suc, Mal, Hip, and Sal-D_6_ (IS) was performed in MRM mode (See also Table S5). The ESI and MS/MS conditions were as followed; capillary voltage 2 kV; source offset 50 V; desolvation temperature 550°C; source temperature 150°C; desolvation gas (N_2_) flow 800 L/h; cone gas flow 150 L/h; nebulizer gas (N_2_) 7.0 bar; collision gas (Ar) 0.25 mL/min. A volume of 5 μL of sample or standard was injected onto an HSS T3 column (2.1 × 100 mm, 1.7 μM column; Waters, Milford, MA, USA) at 40°C with a constant flow rate of 0.4 mL/min. Mobile phases were (A) 0.1% formic acid in Milli-Q water and (B) 0.1% formic acid in acetonitrile (ACN). The gradient program started at 99% A for 0.5 min, decreased A to 90% over 1.0 min, decreased A to 70% over 3.5 min, decreased A to 30% over 3.0 min, and returned to the initial condition over 3.0 min where it was held for 3.0 min. The total runtime was 14 min. The weak and strong needles wash solutions were with 5% ACN and H_2_O/ACN/MeOH/IPA (1:1:1:1), respectively. The quantitative analysis was performed based on external calibration curves of each standard prepared in pooled urine samples (standard addition^18^). The linear ranges of the calibration standards are as following: 59–29,500 ng/ml for Suc; 67–33,500 ng/ml for Mal; 89.5–89,500 ng/ml for Hip, and 14.4–720 ng/ml for Sal-D_6_.

#### GC-MS/MS analysis of non-volatile organic acids

The non-volatile organic acids analyzed by GC-MS/MS were pyruvic acid (Pyr), lactic acid (Lac), oxalic acid (Oxa), malonic acid (Malo), methylmalonic acid (MMA), succinic acid (Suc), fumaric acid (Fum), oxaloacetic acid (OAA), malic acid (Mal), α-ketoglutaric acid (Ket), and citric acid (Cit).

#### Preparation of urine samples

Urine samples were prepared according to Deng et al., (2015) with minor modification (Deng et al., 2015). Briefly, a 100 μL of urine sample was mixed with 250 μL of ACN. The mixture was vortexed at 2,200 rpm for 5 min and centrifuged at 10,000 rpm for 10 min (4°C). Then, 250 μL of the supernatant was collected and transferred to a wine-glass shape GC vial (Agilent, USA) containing 50 μL of 1 mM of myristic-D_27_ acid (Myr-D_27_; IS) dissolved in hexane. The vial was evaporated to dryness (60°C) for 60 min using a vacuum centrifuge (Concentrator plus, Eppendorf, USA). A 50 μL of dichloromethane was added to the sample and evaporated to dryness using the method described above. Subsequently, the sample was derivatized according to the known protocol by Fiehn (2016). Briefly, 50 μL of 40 mg/mL of O-methylhydroxylamine hydrochloride (in pyridine) was added into the dried sample and left at 30°C for 90 min. Subsequently, 50 μL of *N*-methyl-*N*-(trimethylsilyl) trifluoroacetamide containing 1% of trimethylchlorosilane (MSTFA +1% TMCS) was added into the mixtures. The vials were sealed with a screw cap (PTFE/Silicone septa) and incubated at 37°C for 30 min. The GC-MS/MS analysis was performed using gas chromatography (Agilent; 7890B) equipped with a mass spectrometer (Agilent, 7000D) and PAL Auto Sampler System (CTC Analytics AG, Switzerland). A 1 μL of the derivatized sample was injected in a split mode (1:10) at 250°C into a HP-5MS column (30 m, 0.25 mm i.d., Agilent J&W GC column). Helium was used as a carrier gas with a constant flow rate of 1.1 mL/min. The initial oven temperature was set at 80°C, held for 3 min, and programmed from 80°C to 160°C (50°C/min), from 160°C to 340°C (80°C/min), and held for 5 min. The transfer line, ion source (electron ionization; EI), and quadrupole were set as 325°C, 250°C, and 150°C, respectively. The mass spectrometer was operated in a scan mode from 20-650 *m/z* with a data acquisition rate of 3 Hz. MS data were acquired using MassHunter software (version 10.0, Agilent Technologies, USA), utilizing three replicates to calculate the mean and the standard error. The calibration curves used for quantitative were performed using the mixture of non-volatile organic acids at concentrations from 0.02 mM to 2.5 mM. The selected quantifier and qualifier ions are given in Table S6. Qualitative and quantitative analyses were performed on Agilent MassHunter software (version 10.0, Agilent Technologies, USA), and exported into Microsoft Excel for further data processing.

#### Metabolite identification

Metabolite identification in the ^1^H-NMR experiments was achieved through the Statistical Total Correlation Spectroscopy (STOCSY) method and library matching with Human Metabolome Database (HMDB version 3.6, Edmonton, AB, Canada) and in-house chemical shift databases (Nahok et al., 2019) (See also Figure S6A). The identification of chromatographic features detected by GC-MS/MS (See also Figure S6B) and LC-MS/MS (See also Figures S6C and S6D) from urine samples was carried out by comparing their retention times and mass spectrum profiles with the authentic standards (Dunn et al., 2013).

#### Pathway analysis and candidate metabolite

Candidate metabolites previously identified were converted to KEGG compound identifiers and searched against the KEGG pathway (Kanehisa et al., 2020) by the “Search&Color Pathway” tool in the KEGG mapper (https://www.genome.jp/kegg/tool/map_pathway2.html (Kanehisa and Sato, 2020)), which allows multiple mapping operations to be done simultaneously and also highlights pathways and keywords associated with renal injury in LN patients.

### Quantification and statistical analysis

The metabolomics data from ^1^H-NMR (semi-quantitative analysis) were log_2_-transformed while the data obtained from LC-MS/MS and GC-MS/MS (absolute quantification; mM) were normalized to UCr (mM) without further transformation with the normalized result having no unit. The [Pic/Trp] ratio was calculated by the concentration of Pic ([Pic]) divided by the concentration of Trp ([Trp]).

Principal component analysis (PCA) was performed on the dataset for a global view of metabolite profiling using *prcomp()* function in R. Mann-Whitney U test was performed to identify statistically significant metabolites that can differentiate LN from CON groups, and class V from pure class III/IV. False discovery rate (FDR) with Benjamini-Hochberg procedure was applied to calculate adjusted p value from multiple comparisons across all metabolites (Khoomrung et al., 2017). Metabolites with adjusted p value < 0.05 and fold change (FC) ≥ 1.5 were considered as candidate metabolites for further analyses. Significance levels of p value were classified as not statistically significant - ns (p value > 0.05), ∗ (p value < 0.05), ∗∗ (p value < 0.01), ∗∗∗ (p value < 0.001), ∗∗∗∗ (p value < 0.0001).

The ability of [Pic/Trp] ratio and two clinical parameters (eGFR and UPCR) to discriminate all class V from pure class III/IV were comprehensively assessed. Seven logistic regression models were generated from those parameters: 1) UPCR, 2) eGFR, 3) UPCR + eGFR (denoted STD), 4) [Pic/Trp] ratio, 5) UPCR + [Pic/Trp] ratio, 6) eGFR + [Pic/Trp] ratio and 7) STD + [Pic/Trp] ratio. For each model, the validation cohort was used as a training dataset for model optimization through repeated 4-fold cross-validation, whereas the discovery cohort was used as a test dataset for model evaluation. Final model performance was determined by the receiver operating characteristic (ROC) analysis using the pROC R package (Robin et al., 2011). The ROC curve plots the true positive rate against the false-positive rate, from which the area under the ROC curve (AUC) is computed (Uddin et al., 2019). The predictability of each model is represented by AUC values.

## Data Availability

• Metabolomics data are available *via* Mendeley Data https://doi.org/10.17632/srcz7scz67.2. Any additional data that support the study’s findings are available from the lead contact upon request. • This paper does not report the original code. All code utilized was publicly available. • Any additional information required to reanalyze the data reported in this paper is available from the lead contact upon request.

## References

[bib1] Adedeji A.O., Pourmohamad T., Chen Y., Burkey J., Betts C.J., Bickerton S.J., Sonee M., Mcduffie J.E. (2019). Investigating the value of urine volume, creatinine, and cystatin C for urinary biomarkers normalization for drug development studies. Int. J. Toxicol..

[bib2] Åkesson K., Pettersson S., Ståhl S., Surowiec I., Hedenström M., Eketjäll S., Trygg J., Jakobsson P.J., Gunnarsson I., Svenungsson E., Idborg H. (2018). Kynurenine pathway is altered in patients with SLE and associated with severe fatigue. Lupus Sci. Med..

[bib3] Almaani S., Parikh S.V. (2019). Membranous lupus nephritis: a clinical review. Adv. Chronic Kidney Dis..

[bib4] Anders H.-J., Saxena R., Zhao M.-H., Parodis I., Salmon J.E., Mohan C. (2020). Lupus nephritis. Nat. Rev. Dis. Primers.

[bib5] Badawy A.A. (2017). Kynurenine pathway of tryptophan metabolism: regulatory and functional aspects. Int. J. Tryptophan Res..

[bib6] Badawy A.A., Guillemin G. (2019). The plasma [Kynurenine]/[Tryptophan] ratio and indoleamine 2,3-dioxygenase: time for appraisal. Int. J. Tryptophan Res..

[bib7] Bajema I.M., Wilhelmus S., Alpers C.E., Bruijn J.A., Colvin R.B., Cook H.T., D'Agati V.D., Ferrario F., Haas M., Jennette J.C. (2018). Revision of the International Society of Nephrology/Renal Pathology Society classification for lupus nephritis: clarification of definitions, and modified National Institutes of Health activity and chronicity indices. Kidney Int..

[bib8] Cambiaghi A., Ferrario M., Masseroli M. (2016). Analysis of metabolomic data: tools, current strategies and future challenges for omics data integration. Brief. Bioinform..

[bib9] Cellesi F., Li M., Rastaldi M.P. (2015). Podocyte injury and repair mechanisms. Curr. Opin. Nephrol. Hypertens..

[bib10] Chen Y., Guillemin G.J. (2009). Kynurenine pathway metabolites in humans: disease and healthy States. Int. J. Tryptophan Res..

[bib11] Chen Y., Sun J., Zou K., Yang Y., Liu G. (2017). Treatment for lupus nephritis: an overview of systematic reviews and meta-analyses. Rheumatol. Int..

[bib12] Deng M., Zhang M., Sun F., Ma J., Hu L., Yang X., Lin G., Wang X. (2015). A gas chromatography-mass spectrometry based study on urine metabolomics in rats chronically poisoned with hydrogen sulfide. Biomed. Res. Int..

[bib13] Dunn W.B., Erban A., Weber R.J.M., Creek D.J., Brown M., Breitling R., Hankemeier T., Goodacre R., Neumann S., Kopka J., Viant M.R. (2013). Mass appeal: metabolite identification in mass spectrometry-focused untargeted metabolomics. Metabolomics.

[bib14] Fanouriakis A., Kostopoulou M., Cheema K., Anders H.J., Aringer M., Bajema I., Boletis J., Frangou E., Houssiau F.A., Hollis J. (2020). 2019 update of the joint European league against rheumatism and European renal association-European Dialysis and transplant association (EULAR/ERA-EDTA) recommendations for the management of lupus nephritis. Ann. Rheum. Dis..

[bib15] Fernandez-Pol J.A., Bono V.H.,, Johnson G.S. (1977). Control of growth by picolinic acid: differential response of normal and transformed cells. Proc. Natl. Acad. Sci. U S A..

[bib16] Fiehn O. (2016). Metabolomics by gas chromatography-mass spectrometry: combined targeted and untargeted profiling. Curr. Protoc. Mol. Biol..

[bib17] Gaber T., Chen Y., Kraus P.-L., Buttgereit F., Galluzzi L., Rudqvist N.-P. (2019). International Review of Cell and Molecular Biology.

[bib18] Ganguly S., Kumar U., Gupta N., Guleria A., Majumdar S., Phatak S., Chaurasia S., Kumar S., Aggarwal A., Kumar D., Misra R. (2020). Nuclear magnetic resonance-based targeted profiling of urinary acetate and citrate following cyclophosphamide therapy in patients with lupus nephritis. Lupus.

[bib19] Grapov D., Fahrmann J., Wanichthanarak K., Khoomrung S. (2018). Rise of deep learning for genomic, proteomic, and metabolomic data integration in precision medicine. OMICS.

[bib20] Hirayama A., Nakashima E., Sugimoto M., Akiyama S.-I., Sato W., Maruyama S., Matsuo S., Tomita M., Yuzawa Y., Soga T. (2012). Metabolic profiling reveals new serum biomarkers for differentiating diabetic nephropathy. Anal. Bioanal. Chem..

[bib21] Hochberg M.C. (1997). Updating the American College of Rheumatology revised criteria for the classification of systemic lupus erythematosus. Arthritis Rheum..

[bib22] Hocher B., Adamski J. (2017). Metabolomics for clinical use and research in chronic kidney disease. Nat. Rev. Nephrol..

[bib23] Kanehisa M., Furumichi M., Sato Y., Ishiguro-Watanabe M., Tanabe M. (2020). KEGG: integrating viruses and cellular organisms. Nucleic Acids Res..

[bib24] Kanehisa M., Sato Y. (2020). KEGG Mapper for inferring cellular functions from protein sequences. Protein Sci..

[bib25] Khoomrung S., Martinez J.L., Tippmann S., Jansa-Ard S., Buffing M.F., Nicastro R., Nielsen J. (2015). Expanded metabolite coverage of Saccharomyces cerevisiae extract through improved chloroform/methanol extraction and tert-butyldimethylsilyl derivatization. Anal. Chem. Res..

[bib26] Khoomrung S., Wanichthanarak K., Nookaew I., Thamsermsang O., Seubnooch P., Laohapand T., Akarasereenont P. (2017). Metabolomics and integrative omics for the development of Thai traditional medicine. Front. Pharmacol..

[bib27] Korstanje R., Deutsch K., Bolanos-Palmieri P., Hanke N., Schroder P., Staggs L., Brasen J.H., Roberts I.S., Sheehan S., Savage H. (2016). Loss of kynurenine 3-mono-oxygenase causes proteinuria. J. Am. Soc. Nephrol..

[bib28] Levey A.S., Stevens L.A., Schmid C.H., Zhang Y.L., Castro A.F.,3R.D., Feldman H.I., Kusek J.W., Eggers P., van Lente F., Greene T. (2009). A new equation to estimate glomerular filtration rate. Ann. Intern. Med..

[bib29] Limjiasahapong S., Kaewnarin K., Jariyasopit N., Hongthong S., Nuntasaen N., Robinson J.L., Nookaew I., Sirivatanauksorn Y., Kuhakarn C., Reutrakul V., Khoomrung S. (2021). UPLC-ESI-MRM/MS for absolute quantification and MS/MS structural elucidation of six specialized pyranonaphthoquinone metabolites from *Ventilago harmandiana*. Front. Plant Sci..

[bib30] Mor A., Kalaska B., Pawlak D. (2020). Kynurenine pathway in chronic kidney disease: what’s old, what’s new, and what’s next?. Int. J. Tryptophan Res..

[bib31] Nahok K., Li J.V., Phetcharaburanin J., Abdul H., Wongkham C., Thanan R., Silsirivanit A., Anutrakulchai S., Selmi C., Cha'on U. (2019). Monosodium glutamate (MSG) renders alkalinizing properties and its urinary metabolic markers of MSG consumption in rats. Biomolecules.

[bib32] Najafi C.C., Korbet S.M., Lewis E.J., Schwartz M.M., Reichlin M., Evans J., Lupus Nephritis Collaborative Study G. (2001). Significance of histologic patterns of glomerular injury upon long-term prognosis in severe lupus glomerulonephritis. Kidney Int..

[bib33] O'Shea K., Misra B.B. (2020). Software tools, databases and resources in metabolomics: updates from 2018 to 2019. Metabolomics.

[bib34] Okuda S., Nishiyama N., Saito H., Katsuki H. (1998). 3-Hydroxykynurenine, an endogenous oxidative stress generator, causes neuronal cell death with apoptotic features and region selectivity. J. Neurochem..

[bib35] Pawlak K., Mysliwiec M., Pawlak D. (2010). Kynurenine pathway - a new link between endothelial dysfunction and carotid atherosclerosis in chronic kidney disease patients. Adv. Med. Sci..

[bib36] Pertovaara M., Hasan T., Raitala A., Oja S.S., Yli-Kerttula U., Korpela M., Hurme M. (2007). Indoleamine 2,3-dioxygenase activity is increased in patients with systemic lupus erythematosus and predicts disease activation in the sunny season. Clin. Exp. Immunol..

[bib37] Pomyen Y., Wanichthanarak K., Poungsombat P., Fahrmann J., Grapov D., Khoomrung S. (2020). Deep metabolome: applications of deep learning in metabolomics. Comput. Struct. Biotechnol. J..

[bib38] Prodinger J., Loacker L.J., Schmidt R.L., Ratzinger F., Greiner G., Witzeneder N., Hoermann G., Jutz S., Pickl W.F., Steinberger P. (2016). The tryptophan metabolite picolinic acid suppresses proliferation and metabolic activity of CD4+ T cells and inhibits c-Myc activation. J. Leukoc. Biol..

[bib39] Reyes-Thomas J., Blanco I., Putterman C. (2011). Urinary biomarkers in lupus nephritis. Clin. Rev. Allergy Immunol..

[bib40] Rhee E.P., Clish C.B., Ghorbani A., Larson M.G., Elmariah S., Mccabe E., Yang Q., Cheng S., Pierce K., Deik A. (2013). A combined epidemiologic and metabolomic approach improves CKD prediction. J. Am. Soc. Nephrol..

[bib41] Robin X., Turck N., Hainard A., Tiberti N., Lisacek F., Sanchez J.C., Muller M. (2011). pROC: an open-source package for R and S plus to analyze and compare ROC curves. BMC Bioinformatics.

[bib42] Romick-Rosendale L.E., Brunner H.I., Bennett M.R., Mina R., Nelson S., Petri M., Kiani A., Devarajan P., Kennedy M.A. (2011). Identification of urinary metabolites that distinguish membranous lupus nephritis from proliferative lupus nephritis and focal segmental glomerulosclerosis. Arthritis Res. Ther..

[bib43] Savitz J. (2020). The kynurenine pathway: a finger in every pie. Mol. Psychiatry.

[bib44] Sharabi A., Tsokos G.C. (2020). T cell metabolism: new insights in systemic lupus erythematosus pathogenesis and therapy. Nat. Rev. Rheumatol..

[bib45] Tan V.X., Guillemin G.J. (2019). Kynurenine pathway metabolites as biomarkers for amyotrophic lateral sclerosis. Front. Neurosci..

[bib46] Uddin S., Khan A., Hossain M.E., Moni M.A. (2019). Comparing different supervised machine learning algorithms for disease prediction. BMC Med. Inform. Decis. Making.

[bib47] Waikar S.S., Sabbisetti V.S., Bonventre J.V. (2010). Normalization of urinary biomarkers to creatinine during changes in glomerular filtration rate. Kidney Int..

[bib48] Wanichthanarak K., Jeamsripong S., Pornputtapong N., Khoomrung S. (2019). Accounting for biological variation with linear mixed-effects modelling improves the quality of clinical metabolomics data. Comput. Struct. Biotechnol. J..

[bib49] Ward F., Bargman J.M. (2016). Membranous lupus nephritis: the same, but different. Am. J. Kidney Dis..

[bib50] Weening J.J., D'Agati V.D., Schwartz M.M., Seshan S.V., Alpers C.E., Appel G.B., Balow J.E., Bruijn J.A., Cook T., Ferrario F., Fogo A.B. (2004). The classification of glomerulonephritis in systemic lupus erythematosus revisited. J. Am. Soc. Nephrol..

[bib51] Widner B., Sepp N., Kowald E., Ortner U., Wirleitner B., Fritsch P., Baier-Bitterlich G., Fuchs D. (2000). Enhanced tryptophan degradation in systemic lupus erythematosus. Immunobiology.

[bib52] Wishart D.S. (2016). Emerging applications of metabolomics in drug discovery and precision medicine. Nat. Rev. Drug Discov..

[bib53] Wishart D.S. (2019). Metabolomics for investigating physiological and pathophysiological processes. Physiol. Rev..

[bib54] Wishart D.S., Tzur D., Knox C., Eisner R., Guo A.C., Young N., Cheng D., Jewell K., Arndt D., Sawhney S. (2007). HMDB: the human metabolome database. Nucleic Acids Res..

[bib55] Xiang Z.Y., Tang A.G., Ren Y.P., Zhou Q.X., Luo X.B. (2010). Simultaneous determination of serum tryptophan metabolites in patients with systemic lupus erythematosus by high performance liquid chromatography with fluorescence detection. Clin. Chem. Lab. Med..

[bib56] Yoshimura H., Sakai T., Kuwahara Y., Ito M., Tsuritani K., Hirasawa Y., Nagamatsu T. (2009). Effects of kynurenine metabolites on mesangial cell proliferation and gene expression. Exp. Mol. Pathol..

[bib57] Zhao J. (2013). Plasma kynurenic acid/tryptophan ratio: a sensitive and reliable biomarker for the assessment of renal function. Ren. Fail..

[bib58] Zhu M.-R., Fulati Z., Liu Y., Wang W.-S., Wu Q., Su Y.-G., Chen H.-Y., Shu X.-H. (2019). The value of serum metabolomics analysis in predicting the response to cardiac resynchronization therapy. J. Geriatr. Cardiol..

