# Supplementary material for: Predicting lupus membranous nephritis using reduced picolinic acid to tryptophan ratio as a urinary biomarker
Source: iScience. 2021 Oct 25;24(11):103355. doi: 10.1016/j.isci.2021.103355 (PMC8590081; doi:10.1016/j.isci.2021.103355)
Supplement: Document S1. Tables S1–S6 and Figures S1–S6 [file mmc1.pdf]

iScience, Volume *24*

Supplemental information

Predicting lupus membranous nephritis using reduced picolinic acid to tryptophan ratio as a urinary biomarker

Krittima Anekthanakul, Siriphan Manocheewa, Kittiphan Chienwichai, Patcha Poungsombat, Suphitcha Limjiasahapong, Kwanjeera Wanichthanarak, Narumol Jariyasopit, Vivek Bhakta Mathema, Chutima Kuhakarn, Vichai Reutrakul, Jutarop Phetcharaburanin, Atikorn Panya, Natthaporn Phonsatta, Wonnop Visessanguan, Yotsawat Pomyen, Yongyut Sirivatanauksorn, Suchin Worawichawong, Nuankanya Sathirapongsasuti, Chagriya Kitiyakara, and Sakda Khoomrung

Table S1. Top 10 significant metabolites from NMR experiment compared between LN and CON, Related to Results.

Metabolites	Code	Median of CON	Median of LN	Corrected *p*-values	$Log_2(FC)$	Pathway
Pyruvic acid	Pyr	32.15	30.9	0.00010473	-1.25	Glycolysis / Gluconeogenesis; TCA cycle; Pentose phosphate pathway; Pentose and glucuronate interconversions; Ascorbate and aldarate metabolism, etc.
Pyruvatoxime	Pyru	32	32.6	0.00056979	0.6	-
3-Hydroxyisovaleric acid	3-Hia	32.6	31.85	0.00102796	-0.75	Leucine degradation pathway
Citrate	Cit	34	32.1	0.00154642	-1.9	TCA cycle
Unknown1	Unk1	27.85	27	0.00371301	-0.85	-
Hippuric acid	Hip	32.7	30.85	0.00504857	-1.85	Fatty acid oxidation; Phenylalanine metabolism
Trigonelline	Trg	31.6	30.9	0.00999745	-0.7	Nicotinate and nicotinamide metabolism
Choline	Cho	32.4	33.2	0.01539016	0.8	Glycine, serine and threonine metabolism; Glycerophospholipid metabolism; ABC transporters; Cholinergic synapse; Bile secretion; Choline metabolism in cancer
Formate	FA	31.6	30.9	0.03416329	-0.7	Phosphonate and phosphinate metabolism; Pyruvate metabolism; Chloroalkane and chloroalkene degradation; Glyoxylate and dicarboxylate metabolism; Methane metabolism, etc.
Methylsuccinic acid	Msa	31.35	30.75	0.04208852	-0.6	Lipid metabolism pathway; Ethylmalonic encephalopathy

$Log_2(FC)$: Log2 fold change
Cut-off: corrected *p*-values ≤ 0.05 and fold change (FC) ≥ 1.5

Table S2. Significant metabolites from TCA cycle, glycolysis, and fatty acid oxidation measured by GC-MS/MS and LC-MS/MS, Related to Results.

Metabolite	Group1 (gr1)	Group2 (gr2)	Median_gr1	Median_gr2	Corrected *p*-value	Log2(FC)	Pathway
Fum [a]	CON	LN	0.000043	0.000295	0.000004	2.78	TCA
Fum [a]	CON	Pure class III/IV	0.000043	0.000399	0.001961	3.22	TCA
Fum [a]	CON	All class V	0.000043	0.000295	0.000019	2.78	TCA
Fum	Pure class III/IV	All class V	0.000399	0.000295	0.985067	-0.44	TCA
Ket [a]	CON	LN	0.000374	0.001883	0.000069	2.33	TCA
Ket [a]	CON	Pure class III/IV	0.000374	0.003685	0.009341	3.3	TCA
Ket [a]	CON	All class V	0.000374	0.001601	0.000261	2.1	TCA
Ket	Pure class III/IV	All class V	0.003685	0.001601	0.891385	-1.2	TCA
Pyr [a]	CON	LN	0.012419	0.040687	0.000138	1.71	Glycolysis
Pyr [a]	CON	Pure class III/IV	0.012419	0.034767	0.010627	1.49	Glycolysis
Pyr [a]	CON	All class V	0.012419	0.046008	0.00053	1.89	Glycolysis
Pyr	Pure class III/IV	All class V	0.034767	0.046008	0.985067	0.4	Glycolysis
Mal [a]	CON	LN	0.001046	0.006056	0.000165	2.53	TCA
Mal [a]	CON	Pure class III/IV	0.001046	0.007603	0.010627	2.86	TCA
Mal [a]	CON	All class V	0.001046	0.005544	0.000703	2.41	TCA
Mal	Pure class III/IV	All class V	0.007603	0.005544	0.985067	-0.46	TCA
Suc	CON	LN	0.00671	0.009808	0.622329	0.55	TCA
Suc	CON	Pure class III/IV	0.00671	0.006814	0.985067	0.02	TCA
Suc	CON	All class V	0.00671	0.010171	0.505138	0.6	TCA
Suc	Pure class III/IV	All class V	0.006814	0.010171	0.756894	0.58	TCA
Hip	CON	LN	0.007973	0.006587	0.868034	-0.28	Fatty acid oxidation
Hip	CON	Pure class III/IV	0.007973	0.003919	0.532183	-1.02	Fatty acid oxidation
Hip	CON	All class V	0.007973	0.00718	0.985067	-0.15	Fatty acid oxidation
Hip	Pure class III/IV	All class V	0.003919	0.00718	0.622329	0.87	Fatty acid oxidation
Cit	CON	LN	0.010667	0.01328	0.84447	0.32	TCA
Cit	CON	Pure class III/IV	0.010667	0.022693	0.622329	1.09	TCA
Cit	CON	All class V	0.010667	0.01328	0.985067	0.32	TCA
Cit	Pure class III/IV	All class V	0.022693	0.01328	0.639604	-0.77	TCA

Log_2(FC): Log2 fold change; [a]: *p*-value < 0.05

Table S3. Statistical analysis of metabolite ratio in KP measured by LC-MS/MS, Related to Discussion and Figure 7.

Cohort	Metabolite	Median_CON	Median_LN	Corrected *p*-value	Log2(FC)	Regulation
Discovery	[Qui/Trp]	0.4117224	0.289424	0.579737989	-0.508487382	Down-regulation
	[Qui/Kyn]	4.611702	4.28137	0.579737989	-0.107226772	Down-regulation
	[Qui/Kyna]	0.4734866	0.6266545	0.38867491	0.404346649	Up-regulation
	[Qui/3OH-Kyn]	1.3382888	0.8725646	0.071135223	-0.61705563	Down-regulation
	[Qui/Xan][a]	16.58187	43.62865	0.011432946	1.395669119	Up-regulation
	[Qui/3OH-Ant][a,b]	3.287025	7.115084	0.018465494	1.11409836	Up-regulation
	[Qui/Pic][a,b]	85.3	221	0.000123972	1.373428723	Up-regulation
	[Pic/Trp][a,b]	0.004575	0.001345	7.40E-05	-1.766165571	Down-regulation
	[Pic/Kyn][a,b]	0.0726	0.0182	0.00040416	-1.996031098	Down-regulation
	[Pic/Kyna][a,b]	0.00694	0.00212	0.001916113	-1.710871398	Down-regulation
	[Pic/3OH-Kyn][a,b]	0.01575	0.003335	5.81E-05	-2.239593162	Down-regulation
	[Pic/Xan]	0.2195	0.147	0.073185604	-0.578404785	Down-regulation
	[Pic/3OH-Ant]	0.0447	0.03075	0.920809632	-0.539688421	Down-regulation
	[3OH-Ant/Trp] [a]	0.12492478	0.04916831	0.008769097	-1.345259004	Down-regulation
	[3OH-Ant/Kyn] [a]	1.4407236	0.4778836	0.003065141	-1.592062421	Down-regulation
	[3OH-Ant/Kyna]	0.176	0.06685	0.06328312	-1.396575963	Down-regulation
	[3OH-Ant/3OH-Kyn][a,b]	0.4087309	0.0706185	0.000383357	-2.533033233	Down-regulation
	[3OH-Ant/Xan]	6.873319	5.135762	0.416054861	-0.420428571	Down-regulation
	[Xan/Trp] [a]	0.021378728	0.007982765	0.010982725	-1.421215571	Down-regulation
	[Xan/Kyn] [a]	0.26188058	0.09838734	0.013396495	-1.412364486	Down-regulation
	[Xan/Kyna] [a]	0.01929356	0.01224094	0.011432946	-0.656405021	Down-regulation
	[Xan/3OH-Kyn] [a]	0.06828915	0.01835582	0.000123972	-1.895418812	Down-regulation
	[3OH-Kyn/Trp]	0.311815	0.3773916	0.313800462	0.275371981	Up-regulation
	[3OH-Kyn/Kyn]	4.168391	6.24781	0.069220149	0.58385997	Up-regulation
	[3OH-Kyn/Kyna] [a]	0.4281854	0.5898524	0.019082109	0.462118387	Up-regulation
	[Kyna/Trp]	0.674	0.6255	0.247784489	-0.107738707	Down-regulation
	[Kyna/Kyn]	10.858336	7.337162	0.411070581	-0.565508988	Down-regulation
	[Kyn/Trp]	0.06228983	0.07056777	0.9884774	0.180012785	Up-regulation
Validation	[Qui/Trp]	0.1290391	0.1486219	0.327613833	0.203838437	Up-regulation
	[Qui/Kyn]	2.044794	2.245036	0.587016189	0.134783071	Up-regulation
	[Qui/Kyna]	0.1566708	0.1797162	0.08724252	0.197984144	Up-regulation
	[Qui/3OH-Kyn]	0.5198694	0.4694752	0.4506591	-0.14710029	Down-regulation
	[Qui/Xan]	10.15309	13.61418	0.063201676	0.423191224	Up-regulation
	[Qui/3OH-Ant][a]	1.561471	2.411322	0.005519922	0.626918541	Up-regulation
	[Qui/Pic][a,b]	42.5	126	9.43E-09	1.567888987	Up-regulation
	[Pic/Trp][a,b]	0.00321	0.00141	1.29E-07	-1.186878135	Down-regulation
	[Pic/Kyn][a,b]	0.05	0.02165	2.24E-06	-1.20756107	Down-regulation
	[Pic/Kyna][a,b]	0.00341	0.001745	1.15E-06	-0.966544703	Down-regulation
	[Pic/3OH-Kyn][a,b]	0.0124	0.004245	2.36E-07	-1.546503662	Down-regulation
	[Pic/Xan]a	0.271	0.1265	2.26E-05	-1.099155467	Down-regulation
	[Pic/3OH-Ant][a]	0.033	0.0205	0.015496048	-0.686842115	Down-regulation
	[3OH-Ant/Trp]	0.09569796	0.0587917	0.093134694	-0.702875676	Down-regulation
	[3OH-Ant/Kyn]	1.474111	0.8833533	0.057842949	-0.738782694	Down-regulation
	[3OH-Ant/Kyna]	0.0973	0.0805	0.156587861	-0.273451022	Down-regulation

Cohort	Metabolite	Median_CON	Median_LN	Corrected *p*-value	Log2(FC)	Regulation
	[3OH-Ant/3OH-Kyn][a,b]	0.314541	0.1711906	0.005519922	-0.877644597	Down-regulation
	[3OH-Ant/Xan]	7.541411	5.802001	0.366284036	-0.378283933	Down-regulation
	[Xan/Trp]	0.01290831	0.01093963	0.366284036	-0.238736186	Down-regulation
	[Xan/Kyn]	0.192363	0.1802096	0.512029659	-0.094155464	Down-regulation
	[Xan/Kyna]	0.01537393	0.01367895	0.541663183	-0.168528512	Down-regulation
	[Xan/3OH-Kyn]	0.04875554	0.03974699	0.084888556	-0.294720548	Down-regulation
	[3OH-Kyn/Trp]	0.26186	0.2706061	0.392377993	0.047398661	Up-regulation
	[3OH-Kyn/Kyn]	4.295503	5.491273	0.161342796	0.354313557	Up-regulation
	[3OH-Kyn/Kyna]	0.300248	0.391461	0.08724252	0.382713948	Up-regulation
	[Kyna/Trp]	0.862	0.779	0.366284036	-0.146064541	Down-regulation
	[Kyna/Kyn]	13.27589	11.86987	0.443673781	-0.161504446	Down-regulation
	[Kyn/Trp]	0.06065377	0.05939612	0.9541529	-0.030228629	Down-regulation

Log2(FC): Log2 fold change
[a] : *p*-value < 0.05
[b] : Common significant metabolites among cohorts

Table S4. LC-MS/MS conditions for the measurement of metabolites in KP, Related to STAR Methods.

Order	Standard	Code	Retention time (min)	MRM-transition (m/z)	Cone voltage (V)	Collision energy (V)
1	Anthranilic acid	Ant	6.20	138.1>120.0	30	8
2	Cinnabarinic acid	Cin	8.32	301.1>283.1	10	15
3	Kynurenic acid	Kyna	4.46	190.0>144	10	18
4	Kynurenine	Kyn	3.12	209.1>192.1	10	8
5	Picolinic acid	Pic	1.45	124.1>106.0	20	9
6	Quinolinic acid	Qui	1.75	168.0>78.0	10	17
7	Tryptophan	Trp	4.16	205.1>188.1	20	8
8	Xanthurenic acid	Xan	4.10	206.0>160.0	15	20
9	3-hydroxyanthranilic acid	3OH-Ant	3.82	154.1>136.0	20	11
10	3-hydroxykynurenine	3OH-Kyn	2.19	225.2>110.0	25	20
11	Anthranilic acid C_{13} [a]	Ant-C_{13}	6.20	144.1>126.0	30	17

[a] : Internal standard

Table S5. LC-MS/MS conditions for the measurement of succinic acid, malic acid, and hippuric acid, Related to STAR Methods.

Order	Standard	Code	Retention time (min)	MRM-transition (m/z)	Cone voltage (V)	Collision energy (V)
1	Succinic acid	Suc	1.75	117.0>73.0	20	15
2	Malic acid	Mal	0.88	133.0>115.0	25	12
3	Hippuric acid	Hip	3.66	178.0>134.0	20	15
4	Salicylic acid-D_6 [a]	Sal-D_6	5.52	141.0>97.0	15	15

[a] : Internal standard

Table S6. GC-MS/MS analysis of metabolites in TCA cycle and glycolysis metabolites, Related to STAR Methods.

Order	Compound	Code	RT	Quantifier	Qualifier 1	Qualifier 2
1	Pyruvic acid	Pyr	4.91	174	115	158
2	Lactic acid	Lac	4.99	147	117	191
3	Oxalic acid	Oxa	5.46	147	190	73
4	Malonic acid	Malo	5.96	147	233	133
5	Methylmalonic acid	MMA	6.03	147	247	218
6	Succinic acid	Suc	6.80	1147	247	172
7	Fumaric acid	Fum	7.08	245	147	143
8	Oxaloacetic acid	OAA	7.60	290	147	202
9	Malic acid	Mal	8.38	147	233	245
10	α-ketoglutaric acid	Ket	8.86	147	120	156
11	Myristic acid-D_{27} [a]	Myr-D_{27}	9.77	312	120	135
12	Citric acid	Cit	9.79	273	347	363

[a] : Internal standard

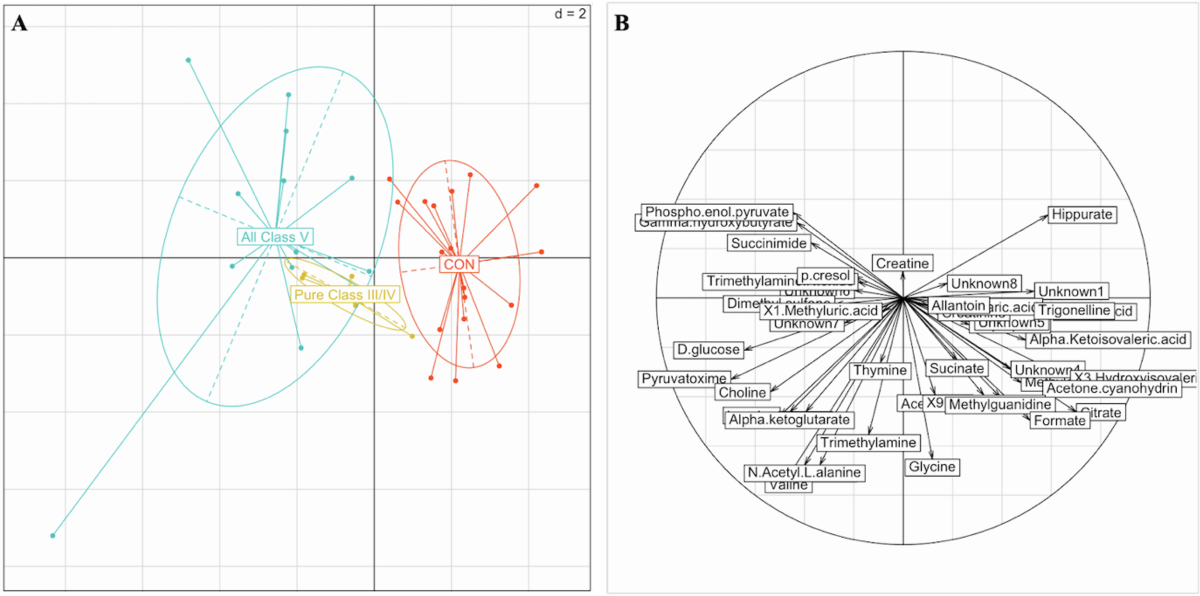

Figure S1. NMR metabolomics data of 38 samples in the discover cohort. Color-coded based on groups and classes of subjects. (A) PCA: score plot of all 38 subjects (each dot represents one subject). (B) PCA: loading plot of metabolites. $R^2X[1] = 0.17$, $R^2X[2] = 0.121$. Note that R^2X represents the explained variance of the component, Related to Results and Discussion.

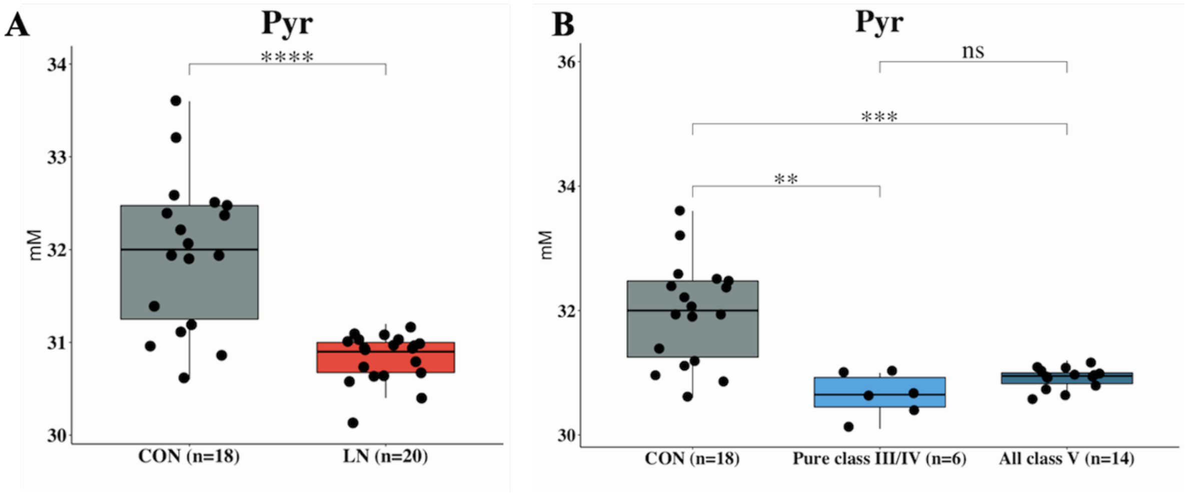

Figure S2. Significant levels of Pyr metabolite from NMR experiment explaining the separation of LN and CON in the discovery cohort. Data are shown as ns (not significant) = *p*-value > 0.05, * = *p*-value < 0.05, ** = *p*-value < 0.01, *** = *p*-value < 0.001, **** = *p*-value < 0.0001. Determined by Mann-Whitney U test, Related to Results.

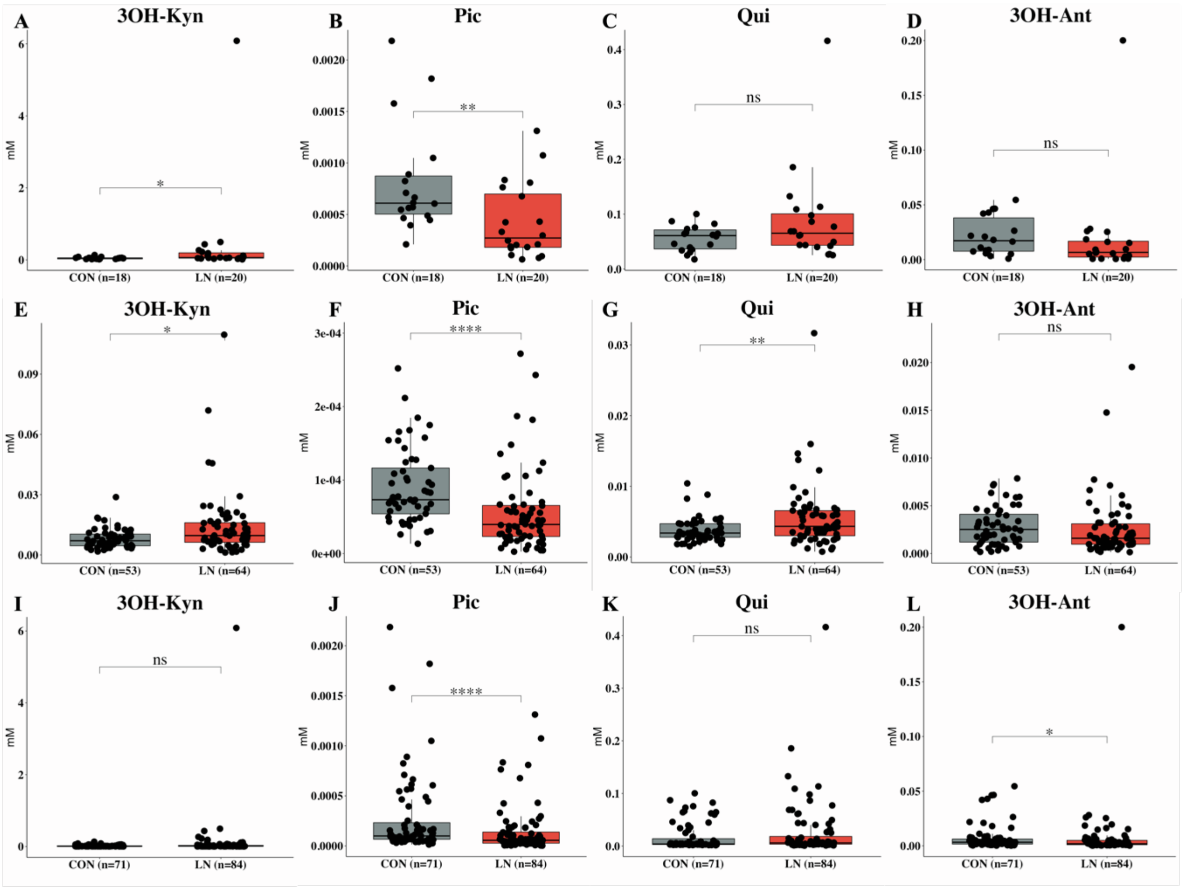

Figure S3. Box plot of significant metabolites in KP for LN prediction from the discovery cohort (A-D), the validation cohort (E-H), and the combined cohort (I-L). Data are shown as ns (not significant) = p-value > 0.05, * = p-value < 0.05, ** = p-value < 0.01, *** = p-value < 0.001, **** = p-value < 0.0001. Determined by Mann-Whitney U test, Related to Results and Discussion.

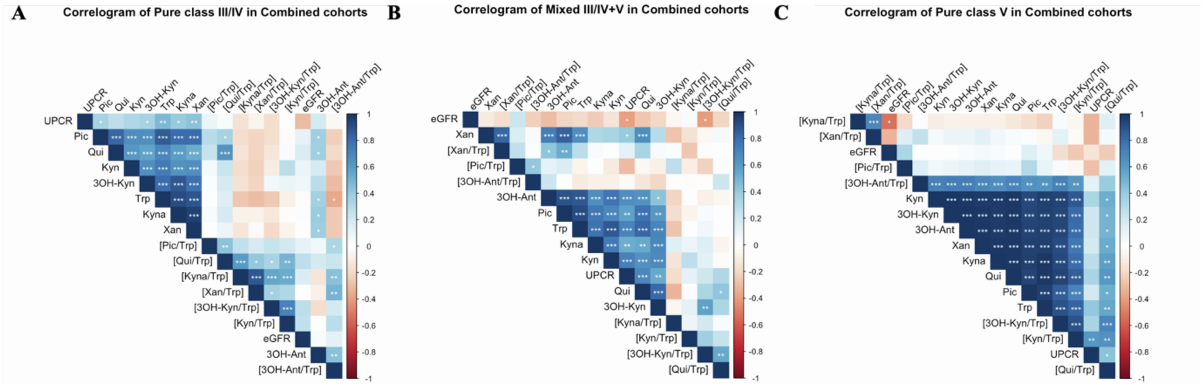

Figure S4. Correlogram among metabolites in (A) Pure class III/IV, (B) Mixed III/IV+V, and (C) Pure class V, Related to Results.

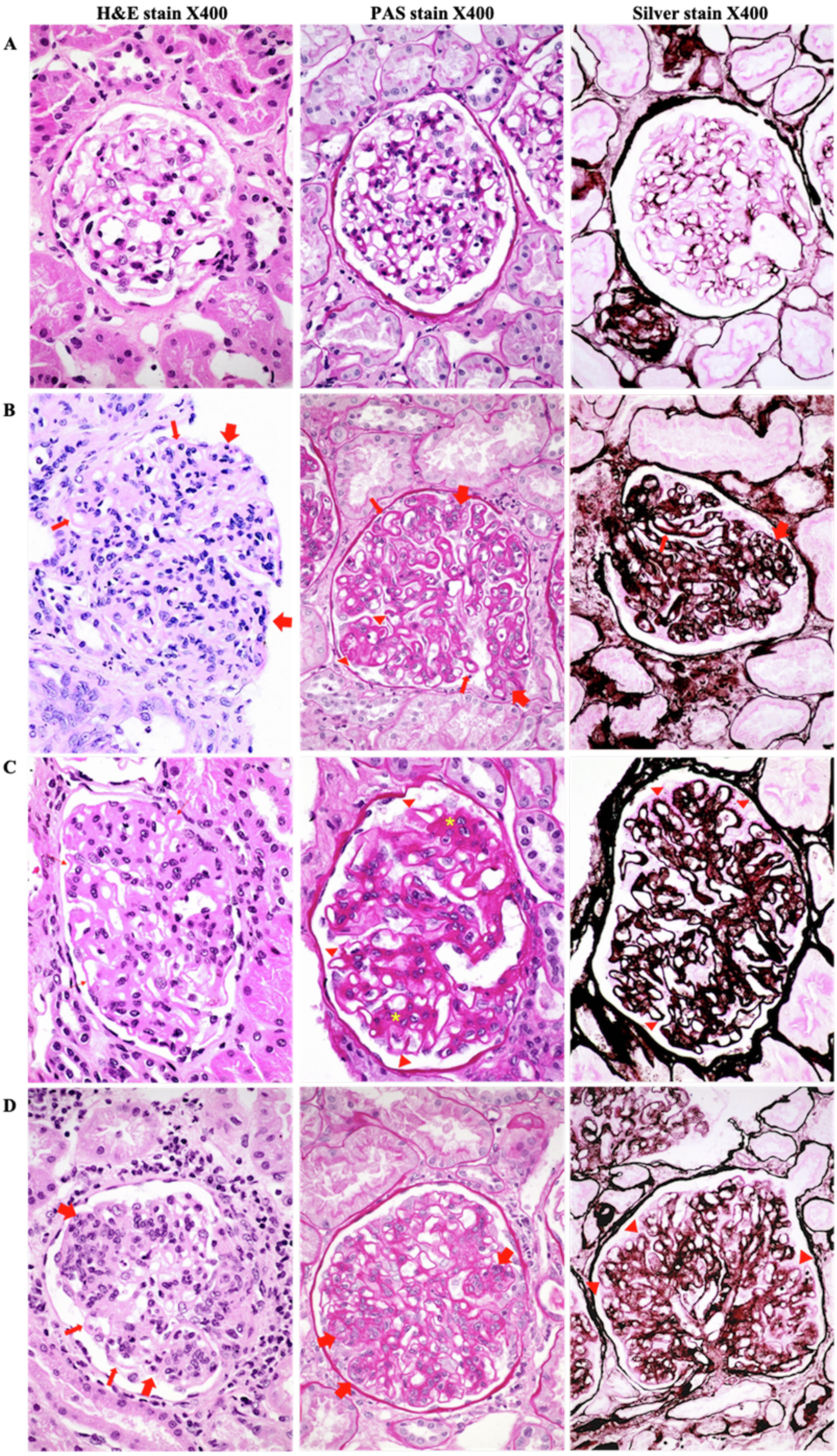

Figure S5. Histopathological sections. (A) Normal glomerulus, (B) Pure class III/IV, demonstrating endocapillary hypercellularity (thick arrow). Wire loop lesions (thin arrow) and hyaline thrombus

(arrow head) represent immune-complex deposits in subendothelium. No spike detected on the silver stain. (C) Pure class V, demonstrating capillary wall thickening on H&E stain (arrow) and mesangial hyperplasia (*) without endocapillary hypercellularity. The PAS and silver staining show spikes formation (arrow head), and (D) Mixed class III/IV +V, demonstrating both features of class III/IV, namely endocapillary hypercellularity on H&E and PAS stain (thick arrow) and class V, namely by thickened capillary wall on H&E stain (thin arrow) and spikes on silver stain (arrow head), Related to STAR Methods.

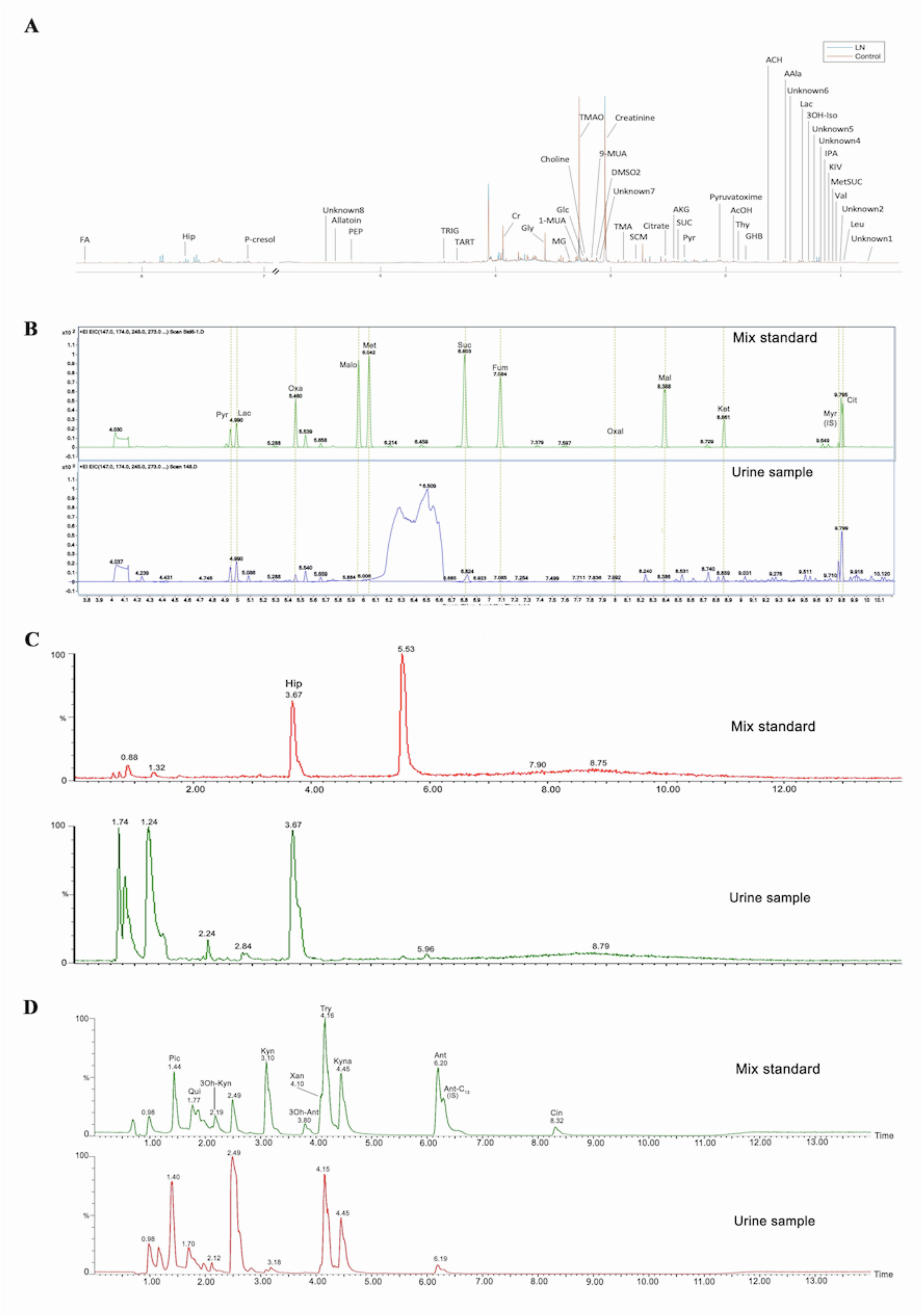

Figure S6. Metabolite detection and identification from different techniques. (A) Metabolites detected by NMR. (B) Metabolites detected by GC-MS/MS. (C) and (D) Metabolites detected by LC-MS/MS, Related to STAR Methods.
